# Animal biosynthesis of complex polyketides in a photosynthetic partnership

**DOI:** 10.1038/s41467-020-16376-5

**Published:** 2020-06-08

**Authors:** Joshua P. Torres, Zhenjian Lin, Jaclyn M. Winter, Patrick J. Krug, Eric W. Schmidt

**Affiliations:** 1 Department of Medicinal Chemistry, University of Utah, Salt Lake City, UT 84112 USA; 2 Department of Biological Sciences, California State University, Los Angeles, CA 90032 USA

**Keywords:** Enzymes, Natural product synthesis, Biosynthesis

## Abstract

Complex polyketides are typically associated with microbial metabolism. Here, we report that animals also make complex, microbe-like polyketides. We show there is a widespread branch of fatty acid synthase- (FAS)-like polyketide synthase (PKS) proteins, which sacoglossan animals use to synthesize complex products. The purified sacogolassan protein EcPKS1 uses only methylmalonyl-CoA as a substrate, otherwise unknown in animal lipid metabolism. Sacoglossans are sea slugs, some of which eat algae, digesting the cells but maintaining functional chloroplasts. Here, we provide evidence that polyketides support this unusual photosynthetic partnership. The FAS-like PKS family represents an uncharacterized branch of polyketide and fatty acid metabolism, encoding a large diversity of biomedically relevant animal enzymes and chemicals awaiting discovery. The biochemical characterization of an intact animal polyketide biosynthetic enzyme opens the door to understanding the immense untapped metabolic potential of metazoans.

## Abstract (web summary)

Complex polyketides are usually produced by microbes, whereas the origin of polyketides found in animals remained unknown. This study shows that sacoglossan animals, such as sea slugs, employ fatty acid synthase-like proteins to produce microbe-like polyketides.

## Introduction

Animals are rich sources of complex polyketides, yet most polyketides are made by microbes^1–3^. Polyketides isolated from animals are sometimes produced by symbiotic bacteria or dietary organisms and not by the animals themselves^4,5^. However, because of these data, it is often claimed that complex natural products such as polyketides must be made by symbiotic bacteria, despite a lack of evidence for most compounds found in nature. Although increasing evidence suggests that animals themselves make some compounds^6–10^, the origin of most polyketides in animals remains unknown. This problem makes it difficult to supply useful animal compounds as drugs and severely constrains our understanding of chemical diversity and the scope of biosynthesis in nature.

**Fig. 1:**
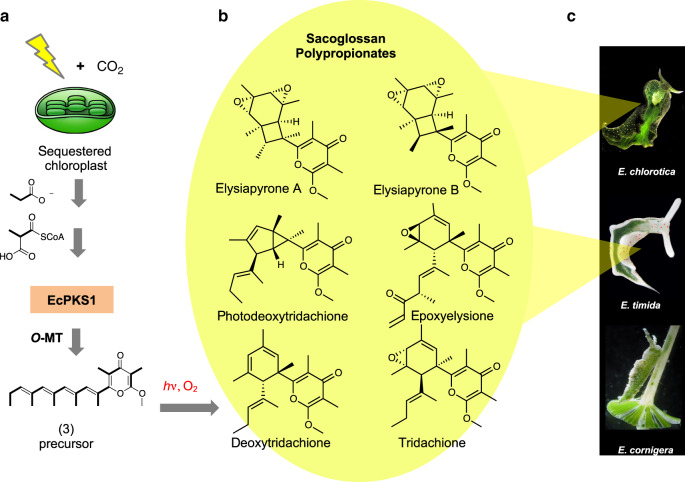
Biogenesis of sacoglossan polypropionates. **a** Previous feeding studies demonstrated that kleptoplasts in the sacoglossans fix carbon that is used to make polypropionates. Propionate is also a precursor, presumably via methylmalonate. A further series of hypothetical steps, including synthesis by a PKS, methylation, and photochemical and oxidation reactions would lead to **b** the known natural polypropionates of diverse structures. These are found in **c** sacoglossan species investigated in this work, including *E. chlorotica* and *E. timida*, but not *E. cornigera*. Photos of *E. chlorotica* and *E. cornigera* by Patrick Krug; *E. timida* by Heike Wägele; used with permission.

The sacoglossan polypropionate pyrones are among the most structurally complex polyketides isolated from animals (Fig. 1). The compounds are representative of a large family of bioactive natural products found in many molluscs around the world, and in all cases the origins of these metabolites are unknown. Sacoglossans consume algae, and some species maintain the stolen algal chloroplasts (kleptoplasts) for weeks or in exceptional cases, for several months^11^. Remarkably, in this symbiosis kleptoplasts are maintained without support from the algal nucleus. Since chloroplasts in plants and algae require many chromosomal genes for their function, this survival has long been a source of mystery and debate^12,13^. The sacoglossan polypropionate pyrones have proposed roles in establishing and maintaining the symbiosis.

Feeding studies in sacoglossans showed that fixed carbon obtained from de novo chloroplast photosynthesis is efficiently incorporated into polypropionates^14^. Labeled propionate is also incorporated^15–17^, suggesting that the mollusc compounds are made via the methylmalonate pathway, which has historically been associated with bacterial metabolism^18^. The polypropionates react via oxidative and photocyclization pathways that are likely non-enzymatic to form the mature natural products^14,19–21^. It is thought that these reactions protect the slugs from damage associated with photosynthesis, and that they may thus be necessary for a photosynthetic lifestyle^19–23^. Thus, the polypropionates are known to originate in these molluscs and to be central to their biology, yet the biochemical source of the compounds remained unknown.

Here, we demonstrate that animals produce microbe-like complex polyketides. Sacoglossans synthesize polypropionate metabolites using a type I iterative polyketide synthase (PKS) encoded on their chromosomes. The PKS resembles the cytoplasmic fatty acid synthase (FAS) found in all animals, yet it is specific for methylmalonyl-CoA over the normal preferred substrate of known animal FAS and enzymes, malonyl-CoA. PKS expression is transcriptionally correlated with acquiring and maintaining the chloroplasts needed to perform photosynthesis, reinforcing previous ideas and data that the polypropionates support kleptoplasty. The FAS-like PKSs form a group of enzymes that branch with animal FAS, unlike characterized PKSs from bacteria, fungi, and animals, which form a distinct family separate from the FAS enzymes. These results greatly increase the scope and complexity of polyketide biosynthesis in animals.

## Results and discussion

### Discovery of polypropionate synthase in sacoglossans

We took an unbiased approach to define the molecular source of polypropionates. To test the bacterial origin hypothesis, we sequenced the metagenomes of polypropionate-containing sacoglossans *Plakobranchus* cf. *ocellatus* “aff. sp. 1”^24^ and *Elysia diomedea*. Good candidate polypropionate synthases were not observed in bacterial metagenomes, suggesting a possible origin in the animals or chloroplasts. We took advantage of recently released genomes and transcriptomes of three sacoglossans^22,25–27^, *Elysia chlorotica*^26,27^, *E. timida*^22,25^, and *E. cornigera*^22,25^, to test this hypothesis. *E. chlorotica* contains polypropionates *ent*-9,10-deoxytridachione and elysione^18^, while *E. timida* contains *ent*-9,10-deoxytridachione and several relatives (Fig. 1, Supplementary Table 1)^28^. Both species obtain fixed carbon from chloroplasts that they maintain for one (*E. timida*) or several (*E. chlorotica*) months. In contrast, no polypropionates have been reported from *E. cornigera*, which only retains chloroplasts for a few days^13^.

**Fig. 2:**
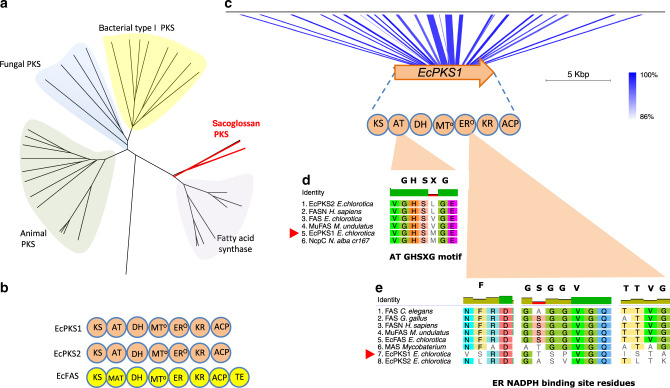
A class of FAS-like PKS enyzmes. **a** Sacoglossan PKSs represent a group separate from the known FAS and PKS proteins from bacteria, fungi and animals. Numbers indicate consensus support in percent. Outgroup DpsA is a type II PKS sequence from bacteria, while the remainder are type I enzymes. **b** The domain architecture of the sacoglossan PKSs, EcPKS1 and EcPKS2 includes ketosynthase (KS), acyltransferase (AT; known as malonyl/acetyltransferase (MAT) in fatty acid biosynthesis), methyltransferase (MT), dehydratase (DH), enoylreductase (ER), ketoreductase (KR) and acyl carrier protein (ACP). This domain architecture is identical to the sacoglossan FAS, EcFAS except for the absence of a C-terminal thioesterase domain. **c** *EcPKS1* is encoded in the genome of *E. chlorotica*. **d** The acyltransferase (AT) domain sequence indicates loading preference for methymalonate while the **e** enoylreductase (ER) domain lacks key NADPH binding site residues making this function inactive.

Based upon recent reports of animals that make polyene fatty acids^6,7^, we suspected that the animals might encode modified FAS or PKS enzymes. Examining the available *E. chlorotica* transcriptomes, we found a number of fragmented transcripts encoding multiple type I FAS enzymes. Reassembly of those transcripts from raw read data revealed four different FAS-like enzymes (Supplementary Table 2). Two of these were the cytoplasmic and mitochondrial FAS enzymes of primary metabolism. The remaining two (EcPKS1 and EcPKS2) formed their own distinct group in a global PKS/FAS tree that included bacterial, fungal, and animal PKS variants (Fig. 2a, Supplementary Fig. 1). *EcPKS1, EcPKS2*, and cytoplasmic *FAS* were encoded in the animal genomes, and not in chloroplasts (Fig. 2c, Supplementary Fig. 2). *EcPKS1* homologs were only found in *E. chlorotica* and *E. timida*, and not in *E. cornigera*, while the other genes were found in all three. This observation was consistent with EcPKS1 being the putative polypropionate synthase.

Like other type I FAS and PKS enzymes, EcPKS1 and EcPKS2 contain multiple domains responsible for product formation (Fig. 2b). Enoylreductase (ER) domains reduce double bonds in the nascent polyketide chain. Because EcPKS1 and EcPKS2 lack functional ER domains, they likely both synthesize polyenes (Fig. 2d)^7,29^. The acyltransferase (AT) domain is responsible for substrate selection, and therefore would be responsible for choosing malonyl-CoA (straight-chain lipids) or methylmalonyl-CoA (methyl-branched lipids)^30^. In bacteria, amino acid residues responsible for AT selectivity are well understood^31^. Since no sequenced animal FASs/PKSs prefer methylmalonyl-CoA, there is no model for AT selectivity in animals. Gratifyingly, EcPKS1, EcPKS2, and several relatives, contained a conserved GHSXGE sequence motif found in bacterial ATs that is known to be important in substrate selection. In bacteria, when in the GHSXGE motif X is methionine, the substrate is methylmalonyl-CoA. In EcPKS1, a GHSMGE sequence was observed, whereas that of EcPKS2 was GHSLGE (Fig. 2e). Comparing these sequence motifs with characterized systems, this implied that EcPKS1 might use methylmalonyl-CoA, while EcPKS2 should use malonyl-CoA. Thus, EcPKS1 had all of the sequence and expression features consistent with the polypropionate synthase.

### Expression and biochemical analysis of EcPKS1

**Fig. 3:**
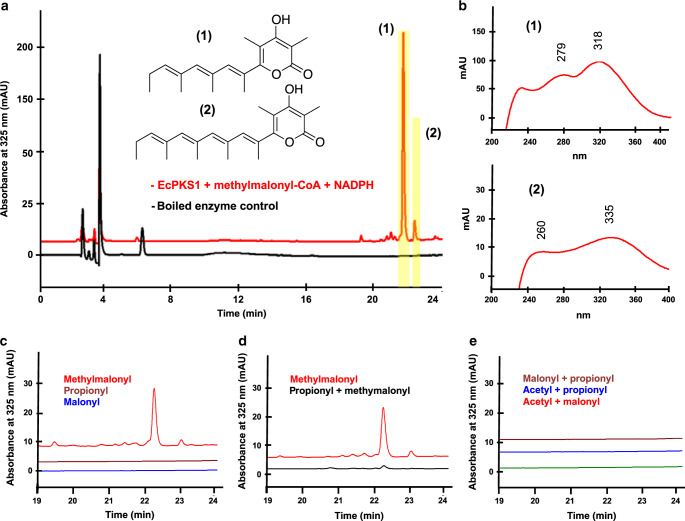
EcPKS1 synthesizes the tridachione precursor. **a** Incubation of EcPKS1 with methylmalonyl-CoA and NADPH resulted in the synthesis of polyene **1** and tridachione precursor **2**. **b** UV spectra of the synthesized products showing features of polyene propionates consistent with the structural assignment. **c** Incubation with other acyl-CoA substrates (malonyl-CoA: m-CoA, propionyl-CoA: p-CoA, methylmalonyl-CoA: mM-CoA) with NADPH did not yield new products. **d** Using propionyl-CoA as the loading molecule inhibited the reaction, as observed in decreased yields. **e** Using different loading and extender units other than methylmalonate did not produce any products. Graphs shown in **a** and **c**–**e** are HPLC-DAD experiments, where the *x*-axis gives the elution time in minutes and the *y*-axis is absorbance units at λ = 325 nm. The plots shown in **b** are UV spectra with the *y*-axis showing absorbance and the *x*-axis showing wavelength in nanometers. Each of these experiments was performed with three biological replicates, each done in triplicate. Representative data are shown.

*EcPKS1* gene was synthesized and expressed solubly as the C-terminally His-tagged construct, in *Saccharomyces cerevisiae* BJ5464 harboring the *npgA* phosphopantetheinyl transferase (PPT) gene (Supplementary Fig. 3)^32^. Incubation of the purified enzyme with methylmalonyl-CoA and NADPH led to a series of new peaks in the chromatographic trace (Fig. 3a, Supplementary Fig. 4). The major peak had a UV spectrum and mass consistent with a triene pyrone that is three carbons smaller than the major natural products of *E. chlorotica* (Fig. 3b). A second, minor tetraene pyrone product had mass and UV features consistent with the putative precursor of tridiachiones and related natural products^15^.

Like other unmethylated, polyunsaturated pyrones, the enzymatic reaction products were challenging to isolate and characterize because of chemical instability. To gain further evidence for the presence of the pyrone structure, we hydrogenated the enzymatic reaction mixture containing the polyene pyrones, and then analyzed the hydrogenation reaction products by gas chromatography-mass spectrometry (GCMS)(Supplementary Fig. 5a–d). This method was chosen because of its predictive value: hydrogenation of the triene should produce four separable stereoisomers, with predictable fragmentation serving to localize methyl groups. Hydrogenation gave four isomers with nearly identical fragmentation patterns around some of the methyl groups, supporting the structural assignment (Supplementary Fig. 5b).

Although some animal FAS/PKS enzymes accept methylmalonyl-CoA in vitro, malonyl-CoA is preferred^33–35^. We sought to determine whether EcPKS1 specifically incorporates methylmalonate, or whether it also uses malonyl-CoA like other enzymes. We incubated EcPKS1 with malonyl-, methylmalonyl-, acetyl-, and propionyl-CoA under various conditions (Fig. 3c–e, Supplementary Fig. 6a–c). These experiments demonstrate that EcPKS1 uses only methylmalonyl-CoA, and not other substrates. Further, our data suggest that other CoA esters compete with methylmalonyl-CoA but are not incorporated into any products, indicating stringent selectivity for methylmalonate. *S*-adenosylmethionine (SAM) is the source of methyl groups in many branched polyketides^29^. We co-incubated SAM with malonyl-CoA and methylmalonyl-CoA in the presence of EcPKS1, showing that SAM is not involved in the formation of the polyketide chain (Supplementary Fig. 6d). Thus, by characterizing EcPKS1 we demonstrate that the clade including animals and fungi can use methylmalonate specifically in lipid biosynthesis.

These data revealed that EcPKS1 is a mollusc polypropionate synthase. In combination with previous experimental evidence, a biosynthetic pathway from CO_2_ to complex polyketides can be defined. Symbiotic chloroplasts fix CO_2_, which is then converted into methylmalonyl-CoA. The animal enzyme EcPKS1 synthesizes the tetraene pyrone, which would be converted to tridachiones and related natural products by methylation, photoisomerization, photorearrangement, and oxidation. These later events may be important in managing stresses associated with photosynthetic activity and are strongly supported by previous synthetic chemistry experiments in which the γ-methylated tetraene pyrone is used as starting material to synthesize the natural products (Supplementary Fig. 8)^36–38^. In these experiments, some steps are known to be UV-catalyzed, while others might still result from rapid thermal rearrangements following photoisomerization.

A remaining mystery involves the in vitro product selectivity observed in our study. The major enzymatic product incorporated six units of methylmalonate, while the minor product incorporated seven. While many mollusc polypropionates contain five or seven equivalents of methylmalonate, none of the reported compounds contains six. Therefore, there is a missing chain length-determining factor. EcPKS1 lacks a thioesterase (TE) domain, which is often important for chain length determination^39^. We attempted to use base to improve offloading of the product^40^, but were unsuccessful, indicating that an as-yet unknown factor governs chain length (Supplementary Fig. 6e).

Overall, the biochemical data demonstrate that the domains of EcPKS1 function similarly to those in PKSs such as fungal lovastatin nonaketide synthase (LNKS)^40^, but with several aspects not previously found in fungal or animal PKS or FAS enzymes. As found in LNKS, both the starter and extender units are the carboxylated CoA esters. Unlike all known fungal and animal enzymes, the EcPKS1 AT domain specifically selects methylmalonyl-CoA, rather than malonyl-CoA, as the starter/extender. In animal FAS, the starter unit is acetyl-CoA, and although malonyl-CoA is the normal extender, the enzymes are tolerant of different substrates in vitro^41^. At each step of chain elongation, the ketoreductase (KR) reduces a β-ketoester to a β-hydroxyester. In the first three-to-four iterations, the DH eliminates the hydroxy group to provide the α,β-unsaturated ester. The ER domain is nonfunctional, so that the resulting product is a polyene. In the final two iterations, the KR does not function, leaving the ketones intact. These steps are somewhat similar to what is found in LNKS when expressed without its *trans*-ER partner^40^, except that SAM is not involved, and methylmalonyl-CoA is the substrate. In addition, EcPKS1 and LNKS are very distantly related in the large PKS/FAS family tree (Fig. 2a).

In many PKS and FAS enzymes, including LNKS^40^, when the enzyme is manipulated a pyrone is often offloaded. This has been speculated to result from a faster kinetic rate for chain extension in comparison to ketoreduction at later steps^40^. Once the β,γ-diketoester is formed, it is spontaneously released from the enzyme via pyrone formation^40^. Here, we observed formation of two different polyketide chain lengths. Chain-length control is complex, as it can be governed by the KS, by chain-release domains, or by diverse factors including auxiliary proteins^42,43^. Here, we do not determine how chain length is controlled. Because the animal EcPKS1 is not part of a biosynthetic gene cluster, or a locus of pathway-specific genes, we cannot easily identify other genes involved in biosynthesis. Therefore, additional enzymes may be involved in chain-length determination, or whether the observed chain-length selectivity obtains in vivo as well.

### EcPKS1 is strongly associated with maintaining photosynthetic chloroplasts

Carefully examining all reports of polypropionates from molluscs, we found that EcPKS1-like products are largely restricted to sacoglossans (Supplementary Table 1). Among these, short, five-propionate products are found in the non-photosynthetic, cerata-bearing slugs, where they may be defensive and are involved in regeneration^44^. The compounds are not very reactive and are not associated with photosynthesis. Sacoglossans can be divided based upon short-term retention (STR), long-term retention (LTR), or no retention (NR) of chloroplasts. Kleptoplasts are digested in less than two weeks in STR species, whereas LTR species can sustain kleptoplasts for one to nine months and may use photosynthesis to survive without feeding for prolonged periods^45,46^.

**Fig. 4:**
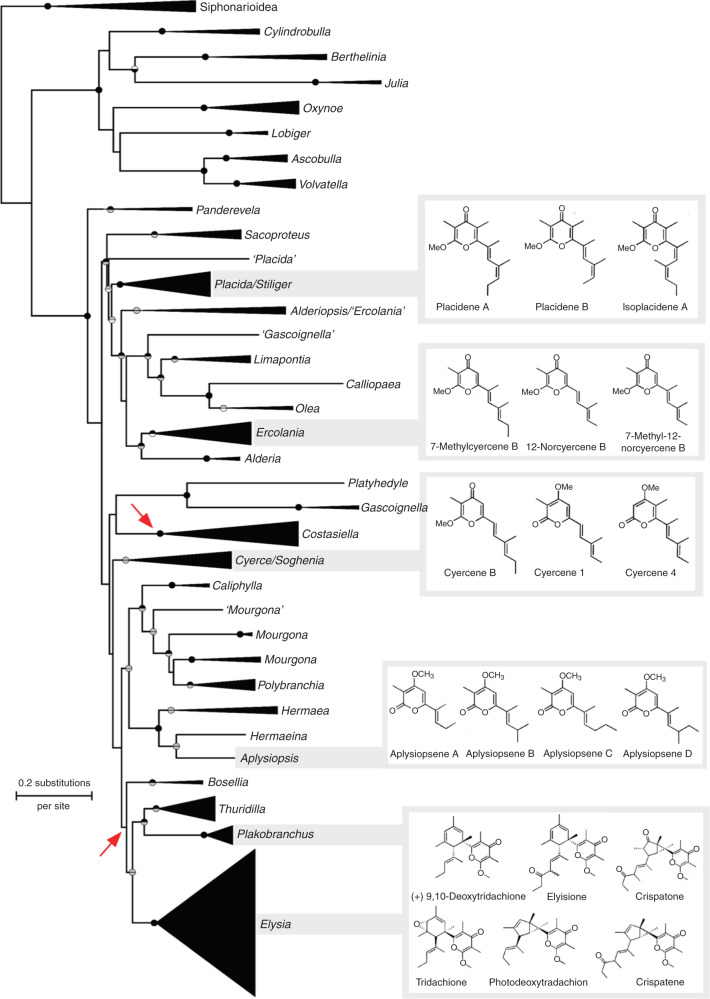
Long-chain polyketides are found only in LTR sacoglossans. Molecular phylogeny of Sacoglossa with genera collapsed, based on maximum likelihood and Bayesian Inference analyses of four gene regions from 219 species^63^. Circles on supported nodes show posterior probability (above branch) and bootstrap percentage (below); black = 1.0 or 100%; grey = significant (≥0.9 or ≥70%) but not complete support; white = not significant. Arrows indicate ancestral nodes in which chloroplast retention and photosynthetic ability likely evolved. Grey bars join a genus to representative polyketides isolated from member species. At the bottom right, a box containing long-chain polypropionates is attached to the LTR sacoglossans.

**Fig. 5:**
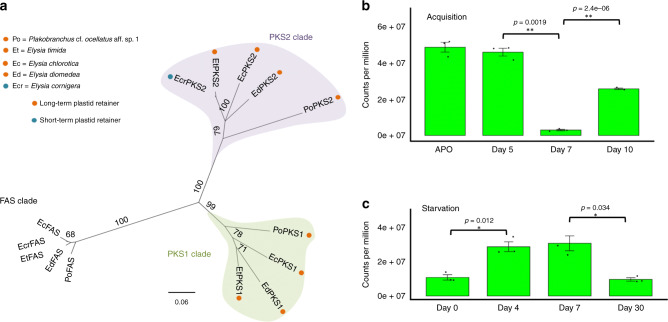
EcPKS1 and relatives are associated with photosynthetic sacoglossans. **a**. EcPKS1 is specific to LTR sacoglossans. A neighbor-joining tree based on the acyltransfersase domain of sacoglossan PKS and FAS proteins made using UPGMA. Numbers indicate consensus support (%). The PKS1 clade contains all LTR organisms (orange dots), while FAS and PKS2 includes the non-LTR *E. cornigera*. **b** Expression of EcPKS1 during development and **c** during starvation. APO indicates the aposymbiotic state, at the transition to the benthic state when chloroplasts are acquired. The expression data thus show that EcPKS1 production is highest as chloroplasts are being acquired, and during starvation its expression increases as photosynthesis becomes more important to animal survival. Each time point has a sample size of *n* = 3 and expressed as mean ± standard error. *P* values (*p* < 0.000****; *p* < 0.001***; *p* < 0.1**; *p* < 0.05*) between time points were done using T test method.

Analyzing reports of chemistry from these animals, we found that the longer seven- and eight-propionate pyrones are restricted to LTR slugs, and were found in all LTR species that have been chemically characterized (Fig. 4). The longer pyrones are UV and singlet oxygen protective, as demonstrated in previous chemical studies^19,20^. To further examine the association of *EcPKS1* with photosynthetic ability, we reinvestigated the *P*. cf. *ocellatus* and *E. diomedea* metagenomes with the hypothesis that *EcPKS1* homologs would be found in each, as these species are also capable of long-term chloroplast retention. Indeed, homologs of *EcPKS1*, *EcPKS2*, and cytoplasmic *FAS* were discovered (Fig. 5a). These were mollusc-encoded, as they came from intron-bearing contigs similar to those encoding their relatives in *E. chlorotica*. Thus, in all of the LTR slugs examined, *EcPKS1* homologs were present, while in the STR slug available, the *EcPKS1* homolog was absent, demonstrating *EcPKS1* is correlated with long-term kleptoplast function.

When we maintained *E. diomedea* in an aquarium, the animals laid eggs, which developed into embryos and hatched. While most defensive compounds in sea slugs such as nudibranchs are highly concentrated in the egg ribbons^47^, polyene pyrones were absent from developing embryos, which lack chloroplasts (Supplementary Fig. 8). Instead, the compounds are highly abundant and concentrated in the juvenile and adult animals, which have fed on algae and are photosynthetic. These data imply that pyrones have a native role other than chemical defense.

We reexamined previous transcriptomes of *E. chlorotica* and *E. timida*, focusing on the described and annotated *EcPKS1* and *EcPKS2*. (The *E. cornigera* transcriptome only contained two timepoints.) In the two animals, the reported transcriptomes show that *EcPKS1* and *EcPKS2* and its homologs are expressed at all time points. *EcPKS1*’s expression pattern was correlated with photosynthesis. In transcriptomes of *E. chlorotica* at various growth stages, *EcPKS1* is highly expressed as the animals metamorphose from the planktonic larval to the benthic juvenile slug stage, just before chloroplasts are acquired (Fig. 5b, c). Previous work on *E. chlorotica* transcription classified the EcPKS1 partial assembly with cluster 3, a group of genes observed to be transcribed during chloroplast acquisition^27^, although the full transcript and function of the protein were not known.

In a previous study of *E. timida* and *E. cornigera* starvation, it was demonstrated that oxidative stress is extremely high in *E. cornigera* and its STR chloroplasts during starvation; the slug quickly dies^22,25^. By contrast, in *E. timida* oxidative stress does not increase upon starvation, and the animals live for extended periods on photosynthesis. The authors conclude that the different response to oxidative stress is crucial to maintaining photosynthesis using naked chloroplasts, and further speculate that pyrones may be the critical antioxidants facilitating this symbiosis. Consistent with this hypothesis, in the transcriptomes reported in their paper, we found expression of the *EcPKS1* ortholog *EtPKS1* is upregulated in *E. timida* as photosynthesis increases, but is downregulated after prolonged starvation, when sequestered chloroplasts have degraded (Fig. 5c). Production of pyrones is thus correlated with the establishment of symbiosis and the increased photosynthesis under initial starvation conditions, but decreases after subsequent kleptoplast degradation.

Overall, our data thus strongly support previous suggestions that pyrones are critical for maintaining long-term photosynthetic activity in sacoglossans by serving antioxidant and photoprotective roles^22,23^. Both previous biological studies and previous chemical studies provide experiments that suggest a photosynthetic-protective role for the pyrones^20,21,23,25^, and here we provide further chemical, genomic, and transcriptomic evidence supporting this idea. In order to prove this role beyond a doubt, further studies using genetic knockout/down would be required.

It is interesting that algal chloroplasts contribute to this by providing the fixed carbon used to produce an animal compound. This may also find biotechnological application. For example, in biofuel development using algae, oxidative stress is a major limitation^48^. Low-level constitutive expression of *EcPKS1* might serve to decrease or eliminate this problem.

### FAS-like PKSs are widespread in animals

**Fig. 6:**
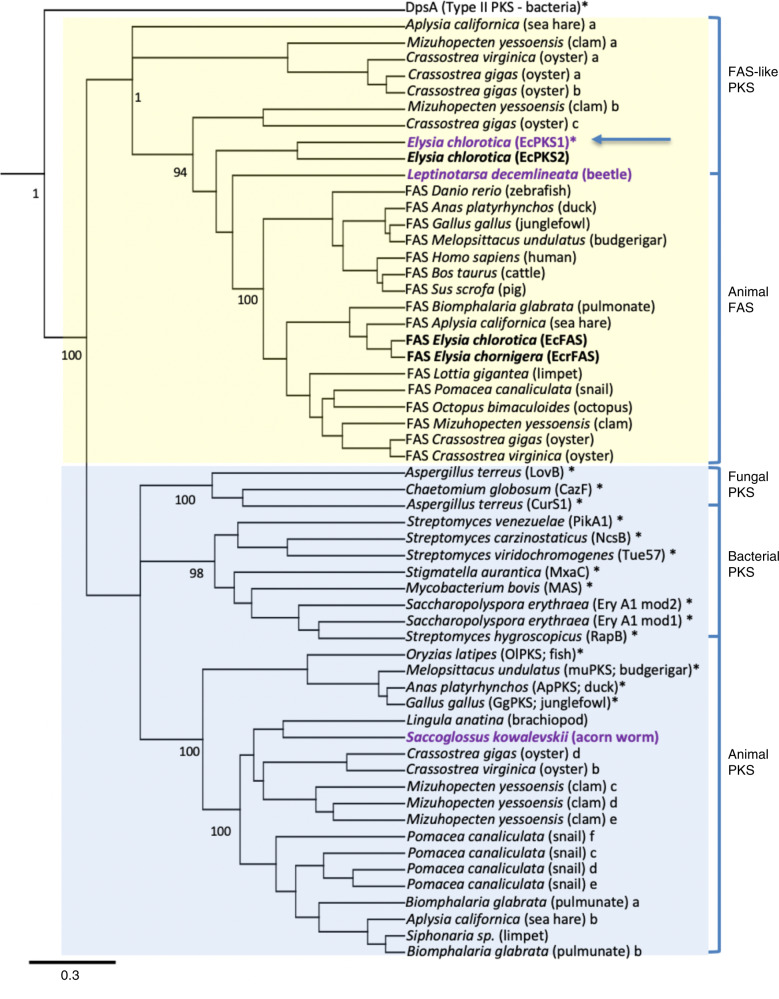
EcPKS1 represents a widespread group of FAS-like PKSs in animals. A phylogenetic tree inferred using Bayesian method was created using protein sequences of ketosynthase domains representing a broad range of characterized and uncharacterized Type I PKSs and FASs. DpsA, a ketosynthase from a Type II PKS, was used as an outgroup. Numbers indicate posterior probability in percent; only key nodes are shown. Asterisk indicates that the protein and / or gene have been characterized. (Animal FAS is also well characterized.) Purple names are for putatively methylmalonate-selective PKSs. In bold are shown sequences discussed in the main text. Letters indicate multiple proteins encoded in the same sequenced genome. The FAS-like PKS group is described in this work.

To explore the extent of potential enzymes and compounds found in the FAS-like PKS family, we mined GenBank to discover many homologs in animals. Some of them may have gone unremarked because they are often poorly assembled in transcriptomes, and many of them are auto-annotated as FAS enzymes. Using these sequences, we generated a taxonomically broader phylogenetic tree based upon the ketosynthase domains of FAS and PKS genes (Fig. 6). Many of the mollusc FAS-like genes, as well as several from other animal phyla, fall in a single large group that includes animal FAS. By contrast, all of the characterized type I PKS genes from bacteria, animals, and fungi belong to a single, large clade. EcPKS1 and EcPKS2 are the only characterized PKS within the large FAS-like PKS cluster. This unexpected branch of lipid metabolism represents a frontier for PKS research.

We sought to examine the extent to which PKSs might be important in molluscs. Polypropionates in marine molluscs are abundant and structurally diverse^2^. Unfortunately, aside from those described in this study, no other sequenced molluscs are known to contain polypropionates. In addition, many molluscs contain linear polyenes, or other PKS-like compounds that based upon structural similarity to EcPKS1 products likely originate in animal metabolism (Supplementary Fig. 9). Examining non-propionate molluscs in GenBank, we found that octopus, sea hares, clams, and oysters contain many FAS-type PKSs that have not been previously noted. However, none of these enzymes falls within the sacoglossan polypropionate group. These data imply that the ability to synthesize complex polyketides is a ubiquitous feature among molluscs, and it may therefore play an important and unrecognized biological role in the animals.

We also sought other animal enzymes that might be methylmalonate specific, since insects and many other animals contain branched polyketides and lipids^10^, and yet the genes that might produce these have not been characterized. So far, we found the methylmalonate motif discovered in EcPKS1 in only two other sequenced animal transcriptomes: from the hemichordate *Saccoglossus kowalevskii* and the beetle *Leptinotarsa decemlineata*. These are distributed in both the known PKS clade and in the FAS-like PKS group. AT phylogeny does not help to discern the substrate preference, since the AT phylogeny mimics that of the underlying KS (Supplementary Fig. 10). Methylmalonate specialization may be relatively uncommon in animals, or perhaps other, undiscovered motifs may yet be important in acyltransferase selectivity.

In summary, EcPKS1 represents biochemical proof that animals can make complex polyketides more typically associated with microbial metabolism. The biosynthesis is part of an elaborate molecular dance in which kleptoplasts from algae fix CO_2_. Fixed carbon is transformed into methylmalonyl-CoA and modified by the mollusc EcPKS1 enzyme to synthesize UV- and oxidation-blocking pyrones that protect the mollusc and its chloroplasts from photosynthetic damage. EcPKS1 is a characterized representative of a widespread family of enzymes in animals.

## Methods

### Genomic DNA extraction, sequencing and assembly

Single live specimens of *Plakobranchus* cf. *ocellatus* “aff. sp. 1” sensu (collected by SCUBA in the Solomon Islands, June 22, 2006; 9° 1.02’S 160° 13.06’ E; appropriate permits obtained following Nagoya Protocol) and *Elysia diomedea* (purchased from www.Liveaquaria.com, Rhinelander, WI, USA) were used in this work. Tissue sections each approximately 0.5 cm^3^ were frozen using liquid nitrogen and ground using a sterile mortar and pestle. Ground tissue was resuspended in lysis buffer (2 mL; 100 mM NaCl, 10 mM EDTA, 1.0% SDS, 50 mM Tris-HCl at pH 7.5) followed by addition of proteinase K (25 µL; 20 mg/mL) and incubated in a water bath at 55 °C until lysis was complete (clear solution). The solution was removed from the water bath and RNAse (2 µL; 20 μg/mL) was added. After 15 min of incubation at 37 °C, saturated KCl (200 µL) was added to the solution. The mixture was incubated in an ice bath for 10 min and then centrifugated at 3739 × *g* at 4 °C. Phenol:chloroform extraction of the supernatant^49^ led to purified DNA. Sequencing was performed at the Huntsman Cancer Institute’s High Throughput Genomics Center, University of Utah. Sacoglossan genomes were sequenced using Illumina HiSeq 2000 sequencer with 350 bp inserts and 125 bp paired-end runs. Raw reads were merged using BBMerge^50^. Non-merged reads were filtered and trimmed using Sickle with the parameters (pe sanger -q 30 –l 40)^51^. The trimmed and merged FASTQ files were assembled using metaSPAdes^52^ with standard parameters in the Center for High Performance Computing at the University of Utah.

### Polypropionate PKS genes in published transcriptomes

Sacoglossan transcriptome reads were downloaded for 19 specimens of *Elysia chlorotica*, *Elysia timida*, and *Elysia cornigera* (Supplementary Table 3). We downloaded the reads from the NCBI Sequence Read Archive (SRA) and assembled them using IDBA-UD^53^. The assembled transcriptomes were queried by Basic Local Alignment Search Tool (BLAST) using the *Homo sapiens* FASN gene (NCBI Accession: NP_0045095.4) involved in human fatty acid metabolism. Putative full-length, assembled FASN-related transcripts were translated using ExPASy translation tool. FASN and related sacoglossan transcripts consist of multiple domains, which were annotated using Conserved Domain Database (NCBI)^54^ and Pfam 32.0 (EMBL)^55^. Geneious 10.2.2 software package was used to perform alignments and tree building. Sequence alignments were made using Multiple Sequence Comparison by Log Expectation^56^, trimmed and realigned. Phlyogenetic trees were constructed using the Unweighted Pair Group Method with the Jukes-Cantor model^57^ and by Bayesian method using MrBayes^58^.

### Construction of EcPKS1 expression plasmid

The plasmid was constructed by homologous recombination in yeast. The predicted *EcPKS1* gene was synthesized in three fragments containing 70-bp overlapping regions (Genewiz). The fragments were designed so that their ends overlapped with the promoter and terminator sites of yeast expression plasmid^40^. The expression vector was linearized using NdeI and PdeII (New England Biolabs) following the manufacturer’s protocol, and the *EcPKS1* fragments and linearized vector were cotransformed into *Saccharomyces cerevisiae* BJ5464 using the S.C. EasyComp Transformation Kit (Sigma). Transformants were plated onto synthetic complete media and selected by transformation to uracil prototrophy. Yeast colonies were combined, and plasmids were isolated using the QIAprep Spin Miniprep Kit (QIAGEN). Plasmids were amplified by transformation into chemically competent *Escherichia coli* DH5α (New England Biolabs), which was plated onto LB agar (10 g/L tryptone, 5 g/L yeast extract, 10 g/L NaCl, agar 20 g/L) with ampicillin (50 μg/mL) and incubated for 24 h at 37 °C. Individual colonies were picked and inoculated in to 5 mL LB broth with ampicillin (50 μg/mL) and incubated at 30 °C with overnight shaking. Plasmids were obtained using the QIAPREP Spin Miniprep Kit (QIAGEN) and screened by digestion with BamHI (New England Biolabs) followed by agarose gel electrophoresis. Plasmid pJTPKS1 containing the expected insert was verified by Sanger sequencing.

### Overexpression and purification of EcPKS1

Plasmid pJTPKS1 was transformed into *Saccharomyces cerevisiae* BJ5464-NpgA (MATα ura3-52 trp1 leu2-Δ1 his3Δ200 pep::HIS3 prb1d1.6 R can1 GAL), which was selected on uracil-deficient agar (1.39 g/L Yeast Synthetic Drop-out Media Supplements without uracil (Sigma-Aldrich), 6.7 g/L Yeast Nitrogen Base (Sigma-Aldrich), 40 mL/L 50% glucose solution, agar 20 g/L) and incubated at 30 °C for 48 h. A single colony of BJ5464-NpgA-EcPKS1 was used to inoculate uracil-deficient broth (5 mL; 1.39 g/L Yeast Synthetic Drop-out Media Supplements without uracil (Sigma-Aldrich), 6.7 g/L Yeast Nitrogen Base (Sigma-Aldrich), 40 mL/L 50% glucose solution) and incubated at 30 °C with shaking at 150 rpm. After 24 h, 1 mL of the culture was used to inoculate 6 × 1 yeast peptone dextrose broth (10 g/L yeast extract, 20 g/L peptone, 20 g/L glucose). The culture was grown at 30 °C under constant shaking at 180 rpm. After 72 h, the cells were pelleted at 3,739 × *g* for 20 min at 4 °C. The cell pellets were resuspended in lysis buffer (50 mM NaH_2_PO_4_, 150 mM NaCl, 10 mM imidazole, pH 8.0) and sonicated on ice at 1-minute intervals until homogenous. The resulting mixture was centrifugated at 28,928 × *g* for 40 min at 4 °C to separate the supernatant from the cell debris. The supernatant was filtered using a 0.45-micron PVDF syringe filter (Millex-HV, Sigma) before adding Ni-NTA and incubating the mixture at 4 °C for 12 h. Soluble EcPKS1 in the lysis buffer was loaded onto Ni-NTA resin in a gravity column. The resin was washed with 25 mL each of 20 mM and 50 mM imidazole in 50 mM Tric-HCl buffer (500 mM NaCl, pH 8.0). The protein was eluted using 3 × 5 L 250 mM imidazole in 50 mM Tris-HCl buffer pH 8.0. 15 mL of eluted EcPKS1 was concentrated to 200 μL, buffer exchanged (15 mL of 50 mM Tris-HCl, 2 mM EDTA, 5 mM DTT, pH 8.0) and further concentrated to 200 μL final volume using Amicon Ultra 100 MWCO centrifugal filters (EMD Millipore). Protein concentration was calculated to be 6.2 mg/L using the Bradford assay with bovine serum albumin (New England Biolabs) as a standard. To check for quality, the expressed EcPKS1 was profiled by size exclusion chromatography on an FPLC (AKTA Go). The Ni-NTA-purified protein was injected onto an S6 column (Superose 6 Increase 10/300 GL, GE Life Sciences) eluting at 0.5 mL/min using buffer (50 mM Tris-HCl, 150 mM NaCl, 1 mM DTT, pH 7.5) (Supplementary Fig. 11B). Fractions were collected every 30 s, and each fraction was concentrated to 20 μL final volume (Amicon Ultra 0.5 mL MWCO 100 KDa, Millipore-Sigma). For each concentrated fraction, 10 μL aliquots were used to run SDS-PAGE (Supplementary Fig. 10B) and the remaining 10 μL aliquots were used to evaluate in vitro enzyme activity. The reaction mixture (in aliquots of 30 μL) was resuspended in methanol (100 μL) and the reconstituted sample (20 μL) was injected onto the UPLCMS (Waters Acquity H- Waters Xevo G2-XS- Q-ToF (Waters) using an Acquity UPLC BEH C18 1.7 μm column (2.1 × 150 m) with a linear gradient of 20–100% solvent B over 10 min (solvent A, H_2_O with 0.05% formic acid; solvent B, MeCN).

### Proteomic analysis of EcPKS1

Proteomic analysis was done at the University of Utah Mass Spectrometry and Proteomics Core. Briefly, EcPKS1 was submitted to the core facility as an excised band from an SDS-PAGE gel, The excised gel was solubilized and digested using trypsin. The tryptic digest was analyzed using quadrupole/time-of-flight hybrid mass spectrometer with electrospray ionization capability (Q-ToF-II, Micromass/Waters). Data was processed through Mascot using customized database according to the predicted amino acid sequence of EcPKS1 (Supplementary Fig. 12).

### In vitro characterization of EcPKS1

All enzymatic reactions described in this manuscript were done using three biological replicates, each of which contained at least three technical replicates, with the exception of the SEC experiment that was done once. Optimization of the enzymatic reaction was done using 100-µL enzyme reaction mixtures consisting of 10 μM EcPKS1, 2 mM NADPH, 1 mM DTT and 2 mM methylmalonyl-CoA buffered using 100 mM sodium phosphate (over a range of pH 6.5–8.0) and incubated at room temperature under dark conditions. After 16 h of incubation, reaction products were extracted twice with ethyl acetate (200 μL). The organic layer was dried using a speed vacuum concentrator and reconstituted with methanol and analyzed by HPLC using a Hitachi Primade HPLC system equipped with a PDA detector and autoinjector. Aliquots (20 μL) of the sample were injected onto a reversed-phase C18 analytical column (Luna C18 4.6 × 100 mm, Phenomenex) with a gradient starting at 20% mobile phase B (H_2_O + 0.1% TFA: MeCN) for 5 min and increased until 100% mobile phase B for 20 min at a rate of 0.7 mL/min. The optimum working pH for EcPKS1 was determined to be 7.5 (Supplementary Fig. 6f). HRMS analysis was performed at the University of Utah’s Mass Spectrometry and Proteomics Core using an Agilent 1290 equipped with an Agilent 1290 FlexCube and Agilent 6350 Accurate Mass Q-TOF dual ESI mass spectrometer. Sample (10 μL) was injected onto a UPHLC C18 column (2.1 × 150 mm Eclipse Poroshell, 1.9 μ ID) set at 30 °C. Elution was done using solvents containing 10 mM ammonium formate (AF) and 0.1% formic acid (FA). Mobile phase A contained water with AF and FA, while mobile phase B contained 95% acetonitrile (aqueous) with AF and FA. The gradient started at 10% mobile phase B for 1 min, increasing to 100% B over 10 min. The flow rate was 0.5 mL/min. Electrospray ionization was done in positive mode using nitrogen as sheath gas at 350 °C flowing at 12 L/min. Capillary and nozzle voltages were set to 3500 V and 1000 V, respectively.

### EcPKS1 product offloading assay

Six 100-µL reaction mixtures containing 2 mM methylmalonyl-CoA, 1 mM DTT, 2 mM NADPH and 10 μM EcPKS1 in 100 mM phosphate buffered at pH 7.5 were incubated in the dark at room temperature for 16 h. After incubation, 1 M NaOH (20 µL) was added to three of the reaction mixtures, which were then incubated at 65 °C for 10 min. Reaction products were extracted into ethyl acetate extraction analyzed by HPLC. For comparison, three control reaction mixtures were made using the same method, but not subjected to NaOH treatment (Supplementary Fig. 6E).

### Methytransferase domain activity assay

Reaction mixtures (100 µL) containing 2 mM malonyl-CoA, 1 mM DTT, 2 mM NADPH, 5 mM S-adenosyl-L-methionine (SAM), 1 mM MgCl_2_ and 10 μM EcPKS1 in 100 mM phosphate buffer (pH 7.5) were incubated in the dark at room temperature. Two parallel enzymatic reaction mixtures containing additional 2 mM methlymalonly-CoA and one without SAM and MgCl_2_ but with 2 mM methylmalonyl-CoA were carried out for comparison. After 16 h of incubation, reaction products were extracted with two volumes of ethyl acetate method and analyzed using HPLC (Supplementary Fig. 6D).

### Acyl substrate specificity assays

Acyl-CoA substrates malonyl-CoA, acetyl-CoA, propionyl-CoA, and methylmalonyl-CoA were purchased from CoALA Biosciences. Enzyme reaction mixtures (100 μL) consisted of enzyme EcPKS1 (10 μM), sodium phosphate pH 7.5 (100 mM), NADPH (2 mM), DTT (1 mM) and acyl-CoA substrates (2 mM). When multiple acyl-CoA substrates were used in a single reaction, they were used at 2 mM each. Reaction mixtures were incubated at room temperature for 16 h and protected from light to avoid photochemical rearrangements (Supplementary Fig. 6).

### Chemical characterization of enzyme reaction products

To generate EcPKS1 products for chemical characterization, fifty enzyme reaction mixtures (100 µL) containing 2 mM methylmalonyl-CoA, 1 mM DTT, 2 mM NADPH and 10 µM EcPKS1 in 100 mM phosphate buffer (pH 7.5) were incubated at room temperature for 48 h with addition of 200 mM NADPH (2 µL) to replenish NADPH after the 24^th^ hour. Enzyme reaction mixtures were pooled and loaded into C18 resin packed in cotton-plugged borosilicate glass Pasteur pipets. The resin was washed twice with water (1 mL) followed by 20% acetonitrile:water (1 mL) before elution with acetonitrile (3 mL). The eluant was dried using a speed vacuum concentrator and reconstituted in methanol 400 µL. Pd/C (0.5 mg) was added to the solution and stirred for 16 h under a H_2_ atmosphere. The mixture was then filtered through Celite to remove Pd/C and dried a using speed vacuum concentrator. GC-MS/MS analysis was performed at the University of Utah’s Metabolomics Core.

GC-MS analysis used an Agilent 7200 GC-QTOF and an Agilent 7693 A automatic liquid sampler. Dried samples were suspended in dry pyridine (100 µL; EMD Millipore) containing *O*-methoxylamine hydrochloride (40 mg/mL; MP Bio) and incubated for one hour at 37 °C in a sand bath. An aliquot (50 µL) of the solution was added to autosampler vials. *N*-Methyl-*N*-trimethylsilyltrifluoracetamide containing 1% chlorotrimethylsilane (60 µL; Thermo) was added automatically via the autosampler and incubated for 30 min at 37 °C. After incubation, samples were vortexed and the prepared sample (1 µL) was injected into the gas chromatograph inlet in the split mode with the inlet temperature held at 250 °C. A 1:1 split ratio was used for analysis. The gas chromatograph had an initial temperature of 60 °C for one minute followed by a 10 °C/min ramp to 325 °C and a hold time of 10 min. A 30-meter Agilent Zorbax DB-5MS with 10 m Duraguard capillary column was employed for chromatographic separation. Helium was used as the carrier gas at a rate of 1 mL/min.

Polyene (**1**): UV (MeCN) λ_max_ 279, 318 nm. HRESIMS *m/z* 289.1814 [M + H]^+^ (calcd for C_18_H_24_O_3,_ 289.1798). GCMS/MS of **1c**: Parent mass *m/z* 366.2548 [M]^•+^ (calcd for C_21_H_38_O_3_Si, 366.2585), Fragments [M]^•+^: *m/z* 73.0474 (calcd for C_3_H_9_OSi, 73.0474), m/z 155.0873 (calcd for C_8_H_15_OSi, 155.0887 *m/z* 211.1141 (calcd for C_11_H_19_O_2_Si, 211.1149) *m/z* 240.1167 (calcd for C_12_H_20_O_3_Si, 240.1177), *m/z* 253.1239 (calcd for C_13_H_21_O_3_Si, 253.1260), *m/z* 351.2322 (calcd for C_20_H_35_O_3_Si, 351.2350).

Tridachione precursor (**2**): UV (MeCN) λ_max_ 260, 355 nm. HRESIMS *m/z* 329.2120 [M + H]^+^ (calcd for C_21_H_28_O_3_, 329.2111).

### Chemical analysis of *E. diomedea*

Chemical extracts were prepared by homogenizing egg ribbons and tissue sections (~10 cm^3^) of *E. diomedea* with acetone (5 mL). The homogenate was centrifuged at 3739 × *g* for 20 min at 4 °C and the supernatant recovered by aspiration. The acetone extract was dried using a rotovap and resuspended in methanol. Extracts were analyzed using a HPLC using a Hitachi Primade HPLC system equipped with a PDA detector and autoinjector. Aliquots (20 µL) were loaded onto a C18 analytical column (Luna C18 4.6 × 100 mm, Phenomenex) with a gradient starting at 30% mobile phase B (H_2_O + 0.1% TFA: MeCN) and 70% mobile phase A (H_2_O) for 5 min and increased until 100% mobile phase B for 25 min at a rate of 1.0 mL/min. Purification of the major polypropionate in *E. diomedea* tissue extract was performed using a semi-preparative HPLC column (Luna 5 u C18 250 × 10 m) at a flowrate of 2.5 mL/min. ^1^H NMR data were obtained using a Varian 500 (^1^H 500 MHz) NMR spectrometer equipped with a 3 mm Nalorac MDBG probe, utilizing residual CDCl_3_ signal for referencing. Natural products, including tridachione, were identified by comparing data to those reported in the literature. A similar procedure was used to isolate and identify products from *P*. cf. *ocellatus*.

### Discovery of EcFAS, EcPKS1, and EcPKS2 analogs

Gene prediction was done using AUGUSTUS^59^. Full-length *EcPKS1*, *EcPKS2*, and *EcFAS* genes were used as queries to search for PKS gene-containing contigs by tblastn search (*E*-value = 1.0 × e^−10^) in *P*. cf. *ocellatus* and *E. diomedea* genomic assembly. Contig hits were then submitted to AUGUSTUS to predict genes based on parameters built using the reported *E. chlorotica* genome assembly and protein sequences^26^. The resulting predicted gene fragments were queried against EcPKS1, EcPKS2 and EcFAS using blastp search (*E*-value = 1.0 × e^−10^) and were ranked according to bitscore and E-value to determine the best hit. The best hits for EcPKS1, EcPKS2 and EcFAS were assigned to each query gene then aligned and assembled to create the predicted PKS and FAS orthologs in *P*. cf. *ocellatus* aff. sp. 1 and *E. diomedea* (PoPKS1, PoPKS2, PoFAS for *P*. cf. *ocellatus* aff. sp. 1 and EdPKS1, EdPKS2 and EdFAS for *E. diomedea*).

### EcPKS1 gene expression analysis

Raw reads were trimmed by Sickle with parameters (pe sanger –q 30 –l 40). Trimmed reads were pooled by species, and de novo assembly for each species was done (EC and ET) using maSPAdes with default parameters. Trimmed raw reads of each specimen were multimapped to the reference transcriptome assembly using Salmon^60^ with parameters (salmon index –t –i index –k 31; salmon quant–index –valudateMappings –libType A –dumpEq –r). The multitude of contigs produced by assembly were hierarchically clustered, and cluster count summarization was performed using Corset 1.04^61^ according to shared read information with parameters (-f true –g –n – salmon+eg_classes). Transcript abundance of clusters (EcPKS1 gene) was estimated using EdgeR^62^ by normalized counts per million. Mean gene expression for *E. chlorotica* (*n* = 3) and *E. timida* (*n* = 3) were calculated on R package (ggpubr) and plotted using ggbarplot function. *P* values (*p* < 0.0001, ****p < 0.001, ***p < 0.1, *p < 0.05*) between time points were calculated using T.test method using stat_compare_means function.

## Supplementary information

Supplementary Information

## Data Availability

All sequences described in this study have been deposited in GenBank (See Supplementary Tables 3 and 4). All other data are available from the corresponding author by reasonable request.
