# Supplementary material for: Animal biosynthesis of complex polyketides in a photosynthetic partnership
Source: Nat Commun. 2020 Jun 8;11:2882. doi: 10.1038/s41467-020-16376-5 (PMC7280274; doi:10.1038/s41467-020-16376-5)
Supplement: Supplementary file 1 — Supplementary Information [file 41467_2020_16376_MOESM1_ESM.pdf]

Supplementary Information

Animal biosynthesis of complex polyketides in a photosynthetic partnership

Joshua P. Torres[1], Zhenjian Lin[1], Jaclyn M. Winter[1], Patrick J. Krug[2], and Eric W. Schmidt[1*]

1. Department of Medicinal Chemistry, University of Utah, Salt Lake City, UT 84112 USA

2. Department of Biological Sciences, California State University, Los Angeles, CA 90032 USA

*Corresponding author: ews1@utah.edu

Supplementary Table 1. Association of long-term chloroplast retention with seven-propionate pyrone compounds.

Species Name (chemistry ref)	Seven-propionate compounds	Plastid retention ability (ref)	EcPKS1 homolog?
Elysia chlorotica (1)	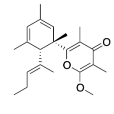 (+) 9,10-deoxytridachione; 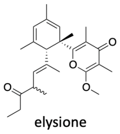 elysione	Up to 9 Months (10)	Yes
Elysia diomedea (2, 3)	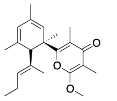 (-) 9,10-deoxytridachione; 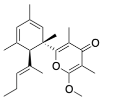 iso-9,10-deoxytridachione; 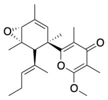 tridachione; 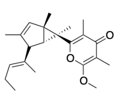 photodeoxytridachione; 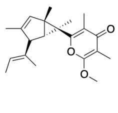 15-norphotodeoxytridachione; 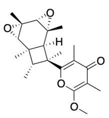 elysiapyrone A; 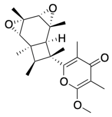 elysiapyrone B; 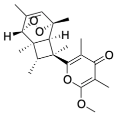 endoperoxide of elysiapyrone	>1 month (11)	Yes
Plakobranchus cf. *ocellatus aff.* sp.1 [a] (4, 5)	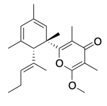 (+) 9,10-deoxytridachione; 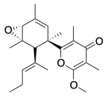 tridachione; 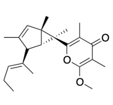 photodeoxytridachione; 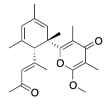 tridachiapyrone I; 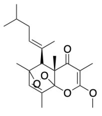 tridachiahydropyrone B; 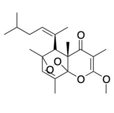 tridachiahydropyrone C; 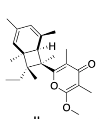 ocellapyrone A; 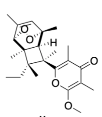 ocellapyrone B	11 months (12-13)	Yes
Elysia timida (6)	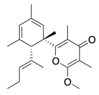 (+) 9,10-deoxytridachione; 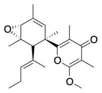 tridachione; 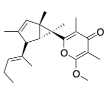 photodeoxytridachione; 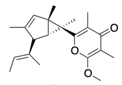 15-norphoto-deoxytridachione	1-2 months (12,14,15)	Yes
Elysia crispata (2, 7)	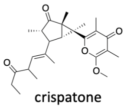 crispatone; 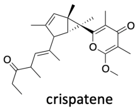 crispatene; 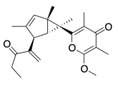 tridachiapyrone E; 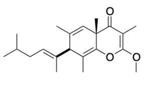 (-) phototridachiahydropyrone; 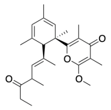 tridachiapyrone A; 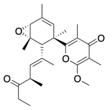 tridachiapyrone C; 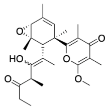 tridachiapyrone D	3 months (16, 17)	Yes
Elysia viridis (8)	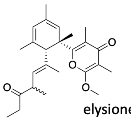 elysione; 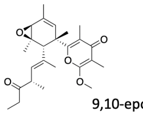 9,10-epoxyelysione	1-2 months (14, 18-20)	ND[b]
Elysia patagonica (9)	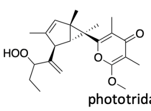 phototridachiapyrone J	ND[b]	ND[b]

[a]congeneric species identified as *P. ocellatus*; [b]ND, not determined

Supplementary Table 2. Genes, proteins and GenBank accession numbers in this study.

Accession number	Species name	Common name	Type	Designation[a]
see table S4	*Elysia chlorotica*	sacoglossan	FAS-PKS	EcPKS1
see table S4	*Elysia chlorotica*	sacoglossan	FAS-PKS	EcPKS2
see table S4	*Elysia chlorotica*	sacoglossan	FAS	
see table S3	*Elysia timida*	sacoglossan	FAS-PKS	EtPKS1
see table S3	*Elysia timida*	sacoglossan	FAS-PKS	EtPKS2
see table S3	*Elysia timida*	sacoglossan	FAS	
see table S3	*Elysia cornigera*	sacoglossan	FAS-PKS	EcorPKS2
see table S3	*Elysia cornigera*	sacoglossan	FAS	
see table S4	*Plakobranchus* cf. *ocellatus* aff. sp.1	sacoglossan	FAS-PKS	PoPKS1
see table S4	*Plakobranchus* cf. *ocellatus* aff. sp.1	sacoglossan	FAS-PKS	PoPKS2
see table S4	*Plakobranchus* cf. *ocellatus* aff. sp.1	sacoglossan	FAS	
see table S4	*Elysia diomedea*	sacoglossan	FAS-PKS	EdPKS1
see table S4	*Elysia diomedea*	sacoglossan	FAS-PKS	EdPKS2
see table S4	*Elysia diomedea*	sacoglossan	FAS	
XP_012937246.1	*Aplysia californica*	sea hare	FAS-PKS	a
XP_005094185.1	*Aplysia californica*	sea hare	FAS	
XP_012938829.1	*Aplysia californica*	sea hare	PKS	b
XP_011414220.1	*Crassostrea gigas*	oyster	FAS-PKS	a
EKC18005.1	*Crassostrea gigas*	oyster	FAS-PKS	a
EKC24795.1	*Crassostrea gigas*	oyster	FAS-PKS	d
EKC30214.1	*Crassostrea gigas*	oyster	FAS	
XP_019926964.1	*Crassostrea gigas*	oyster	PKS	d
XP_021358014.1	*Mizuhopecten yessoensis*	clam	FAS-PKS	a
XP_021350562.1	*Mizuhopecten yessoensis*	clam	FAS-PKS	b
XP_021358822.1	*Mizuhopecten yessoensis*	clam	FAS	

XP_021367770.1	*Mizuhopecten yessoensis*	clam	PKS	c
XP_021339639.1	*Mizuhopecten yessoensis*	clam	PKS	d
XP_021371584.1	*Mizuhopecten yessoensis*	clam	PKS	e
XP_022286280.1	*Crassostrea virginica*	oyster	FAS-PKS	a
XP_022314347.1	*Crassostrea virginica*	oyster	FAS	
XP_022331329.1	*Crassostrea virginica*	oyster	PKS	b
PVD38683.1	*Pomacea canaliculata*	snail	FAS	
PVD27401.1	*Pomacea canaliculata*	snail	PKS	a
XP_025100500.1	*Pomacea canaliculata*	snail	PKS	b
XP_025087737.1	*Pomacea canaliculata*	snail	PKS	c
XP_025087737.1	*Pomacea canaliculata*	snail	PKS	d
XP_025091882.1	*Pomacea canaliculata*	snail	PKS	e
XP_013396153.1	*Lingula anatina*	brachiopod	PKS	
XP_009064332.1	*Lottia gigantea*	limpet	FAS	
XP_019926964.1	*Lottia gigantea*	limpet	PKS	
XP_013076947.1	*Biomphalaria glabrata*	pulmonate	FAS	
XP_013068917.1	*Biomphalaria glabrata*	pulmonate	PKS	a
XP_013079084.1	*Biomphalaria glabrata*	pulmonate	PKS	b
XP_005221054.2	*Bos taurus*	cattle	FAS	
NP_001093400.1	*Sus scrofa*	pig	FAS	
NP_0045095.4	*Homo sapiens*	human	FAS	
XP_009305081.1	*Danio rerio*	zebrafish	FAS	
NP_001297727.1	*Anas platyrhynchos*	junglefowl	FAS	
NP_027306388.1	*Anas platyrhynchos*	duck	PKS	ApPKS
NP_990486.2	*Gallus gallus*	chicken	FAS	
XP_0151537546.1	*Gallus gallus*	chicken	PKS	GgPKS
XP_006815698.1	*Saccoglossus kowalevskii*	acorn worm	PKS	PKS
XP_023011970.1	*Leptinotarsa decemlineata*	beetle	FAS-PKS	

XP_005144267.1	*Melopsittacus undulatus*	budgerigar (bird)	FAS	
XP_005153144.1	*Melopsittacus undulatus*	Budgerigar (bird)	PKS	MuPKS
NP_001295960.1	*Oryzias latipes*	fish	PKS	OlPKS
AAD39830.1	*Aspergillus terreus*	fungi	PKS	LovB
XP_001216280.1	*Chaetomium globosum*	fungi	PKS	CazF
AGC95324.1	*Aspergillus terreus*	fungi	PKS	CurS1
AAK57187.1	*Stigmatella aurantiaca*	bacteria	PKS	MxaC
WP_031702657.1	*Mycobacterium bovis*	bacteria	PKS	MAS
Q9ZGI5.1	*Streptomyces venezuelae*	bacteria	PKS	PikA1
AAM77986.1	*Streptomyces carzinostaticus*	bacteria	PKS	NcsB
AAK83194.1	*Streptomyces viridochromogenes*	bacteria	PKS	Tue57
WP_021341330.1	*Saccharopolypora erythraea*	bacteria	PKS	Ery A1 M1
WP_021341330.1	*Saccharopolypora erythraea*	bacteria	PKS	Ery A1 M2
RLV77292.1	*Streptomyces hygroscopicus*	bacteria	PKS	RapB
WP_100109102.1	*Streptomyces peucetius*	bacteria	PKS	DpSA

[a]Designation refers to the names in Figure 6.

Supplementary Table 3. Sacoglossan genomes and transcriptomes used in this study.

Species Name	BioProject Number	Life Stage	Accession Number	Data Type
Elysia chlorotica	PRJNA484060	benthic slug	RQTK01	whole genome sequence
Elysia chlorotica	SRX3859215	aposymbiotc	SRR6911218	transcriptome
Elysia chlorotica	SRX3859214	aposymbiotc	SRR6911219	transcriptome
Elysia chlorotica	SRX3859212	aposymbiotc	SRR6911221	transcriptome
Elysia chlorotica	SRX3859207	10 day old	SRR6911228	transcriptome
Elysia chlorotica	SRX3859207	10 day old	SRR6911226	transcriptome
Elysia chlorotica	SRX3859204	10 day old	SRR6911229	transcriptome
Elysia chlorotica	SRX3859213	5 day old	SRR6911220	transcriptome
Elysia chlorotica	SRX3859210	5 day old	SRR6911223	transcriptome
Elysia chlorotica	SRX3859211	5 day old	SRR6911222	transcriptome
Elysia chlorotica	SRX3859208	5-7 day old	SRR6911225	transcriptome
Elysia chlorotica	SRX3859209	5-7 day old	SRR6911224	transcriptome
Elysia chlorotica	SRX3859206	5-7 day old	SRR6911227	transcriptome
Elysia cornigera	SRX707504	benthic slug	SRR1582544	transcriptome
Elysia cornigera	SRX707508	benthic slug	SRR1583663	transcriptome
Elysia timida	SRX707526	benthic slug	SRR1582569	transcriptome
Elysia timida	SRX707523	benthic slug	SRR1582566	transcriptome
Elysia timida	SRX707519	benthic slug	SRR1582563	transcriptome
Elysia timida	SRX707518	benthic slug	SRR1583665	transcriptome
Elysia timida	SRX707517	benthic slug	SRR1583664	transcriptome

Supplementary Table 4. Deposited sequence data generated in this study.

Animal Species	Gene name	Accession Number
Elysia chlorotica	EcPKS1	MT348433
Elysia chlorotica	EcPKS2	MT348434
Elysia chlorotica	EcFAS	MT348432

Animal	Gene name	Accession Number
Plakobranchus ocellatus	PoPKS1_AT	PRJNA610421
Plakobranchus ocellatus	PoPKS2_AT	PRJNA610421
Plakobranchus ocellatus	PoFAS_AT	PRJNA610421
Elysia diomedea	EdPKS1_AT	PRJNA610425
Elysia diomedea	EdPKS2_AT	PRJNA610425
Elysia diomedea	EdFAS_AT	PRJNA610425

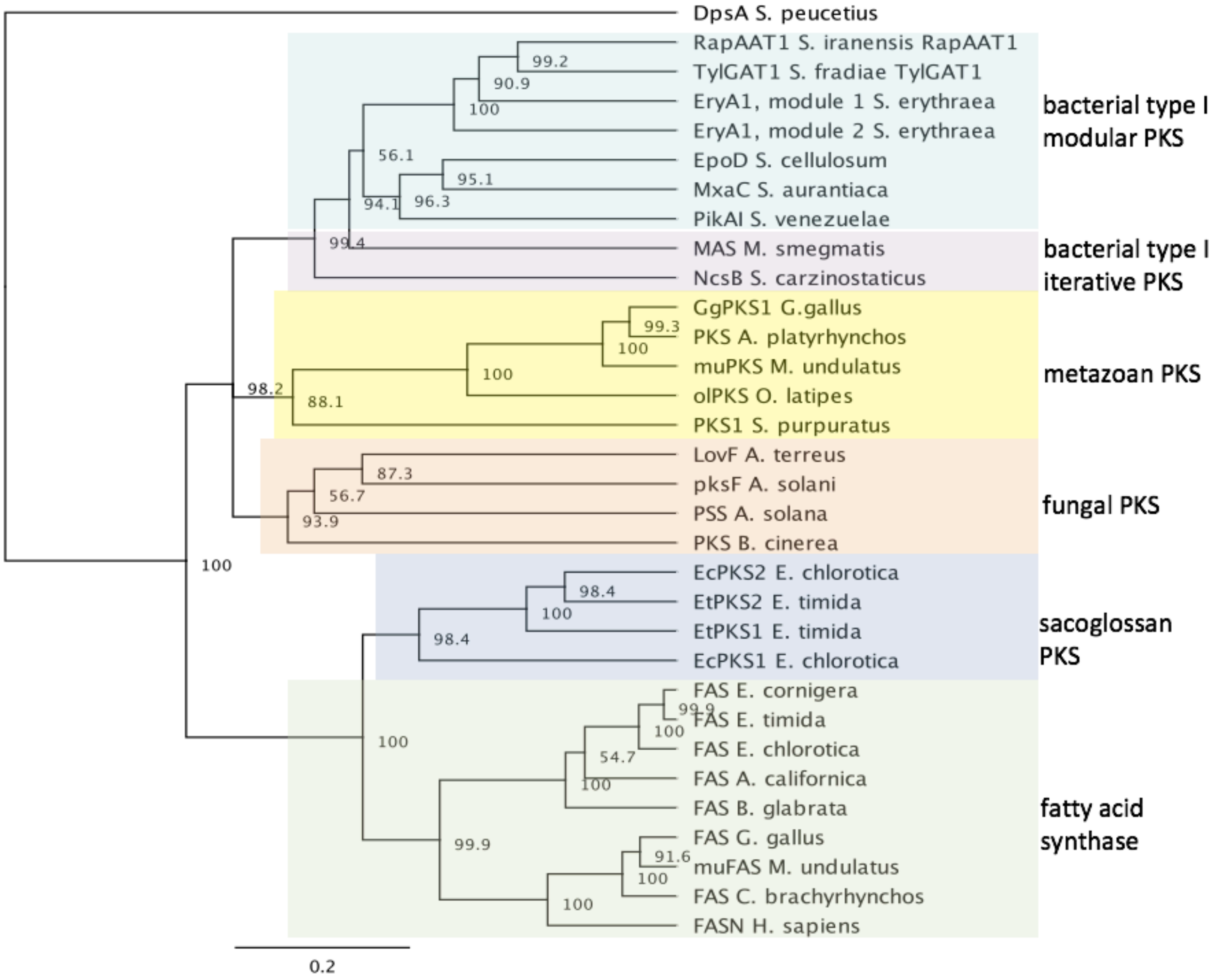

Supplementary Figure 1. **Expansion of phylogenetic tree from Fig. 2A, showing details.** The tree is based on an alignment of ketosynthase domains of sacoglossan polyketide synthases with other known Type I PKS and FAS from fungi, bacteria, and animals. DpsA, a from a Type II PKS from *Streptomyces peucetius* is used as an outgroup. This is a neighbor-joining tree made using UPGMA model as described in Methods. Numbers indicate consensus support in percent.

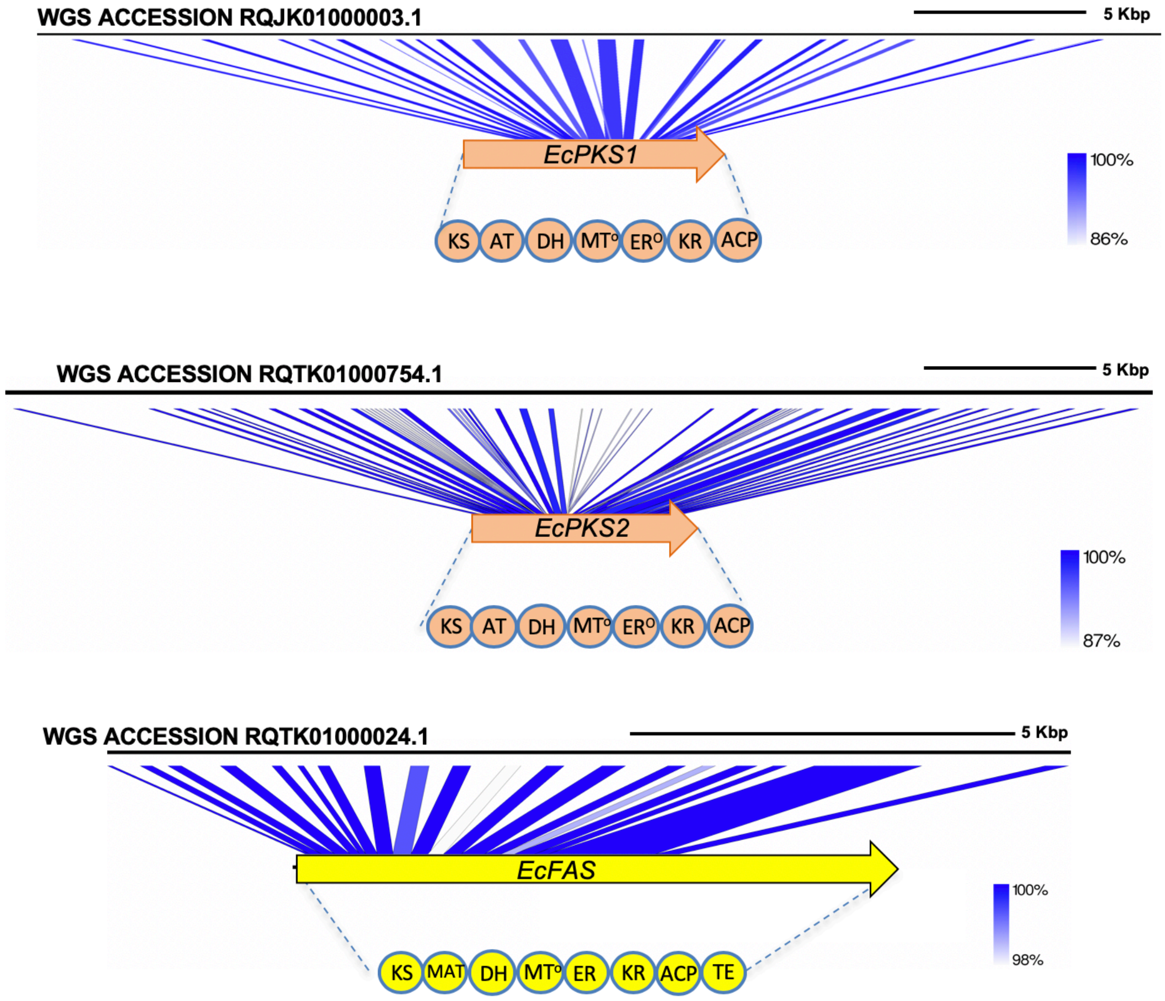

Supplmentary Figure 2. PKS and FAS genes are encoded in the *Elysia chlorotica* chromosome. We reassembled the transcriptome raw reads encoding *EcPKS1*, *EcPKS2* and *EcFAS* in order to generate full-length transcripts. The transcripts were then mapped back to the genome from BioProject PRJNA484060. Flanking the regions that are shown are multiple mollusc-specific genes, demonstrating that the mapped regions represent portions of the animal chromosome. Arrows indicate transcripts, with the underlying bubbles representing individual domains from PKS and FAS proteins. The arrows are connected by blue lines representing exons mapped back to the chromosomes, which are shown as black lines.

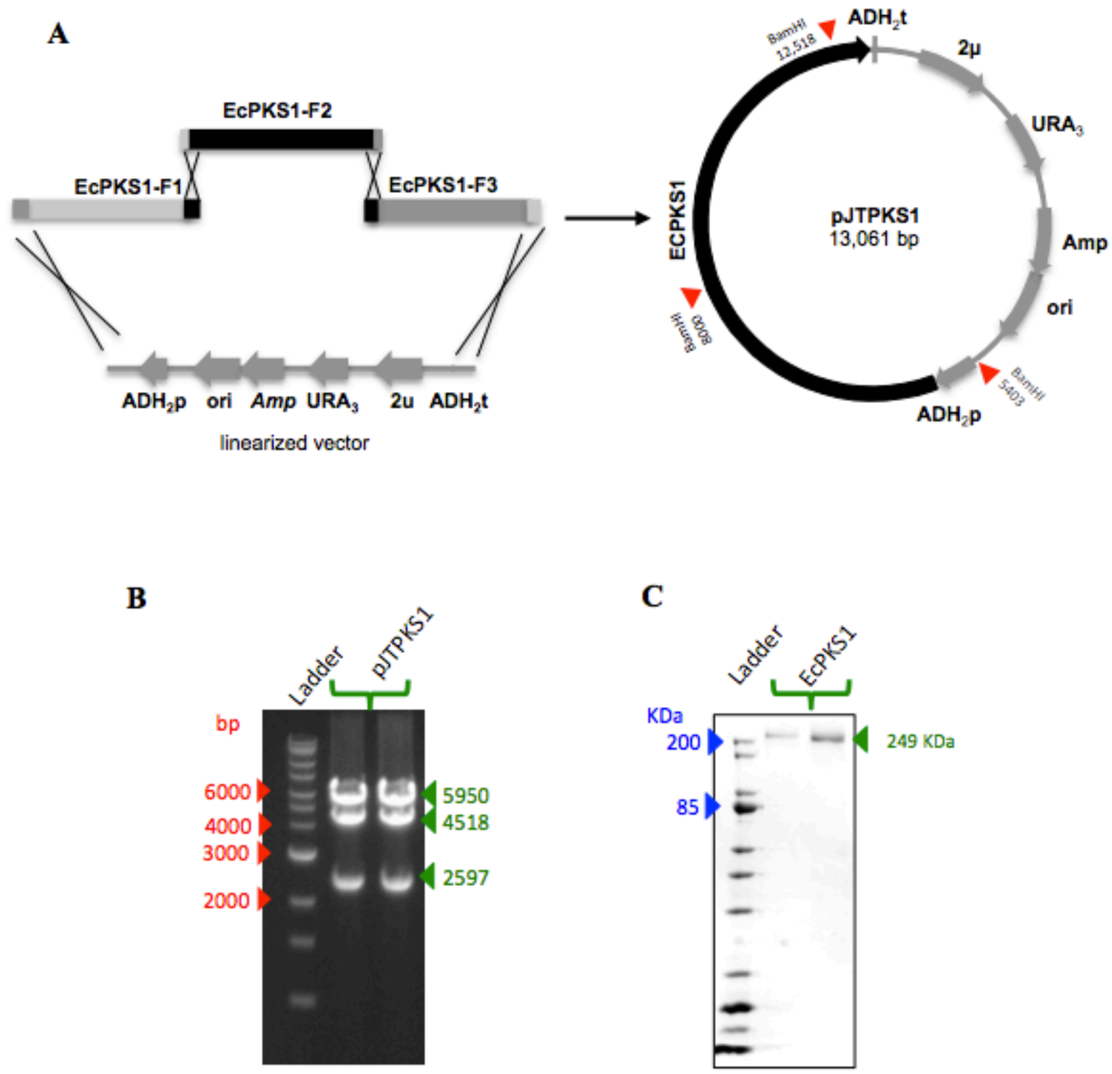

Supplementary Figure 3. **Assembly, expression, and purification of EcPKS1**. **a** Vector pJPTPKS1 was constructed using synthesized fragments of *EcPKS1* and the parent vector[39]. **b** Yeast homologous recombination generated the vector, which was analyzed using restriction fragment length polymorphism analysis with BamHI. **c** SDS-PAGE of expressed and purified C-terminal His-tagged EcPKS1 (249 KDa). The protein was made using *Saccharomyces cerevisiae* BJ5464-NpgA, which encodes a chromosomal pantetheinyltransferase that activates the acyl carrier protein. This protein was independently expressed with similar results a total 7 times.

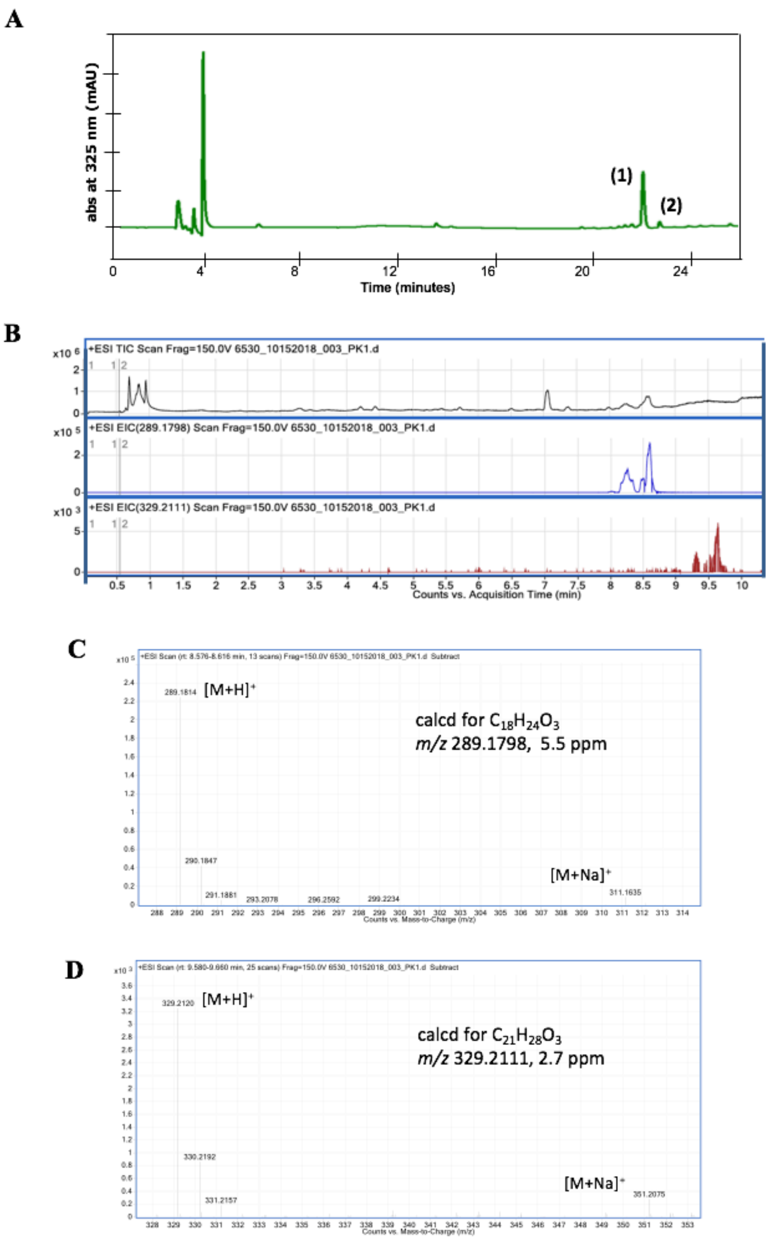

Supplementary Figure 4. **Synthesis of polyene products 1 and 2.** Following reaction of methylmalonyl-CoA with EcPKS1, the products were analyzed by HPLC-DAD and UPLC-MS. **a** HPLC-DAD trace at 325 nm showing the polyene products **1** and **2**. The UV spectra from each peak is shown in Fig. 3B. **b** Total ion chromatogram and extracted ion chromatograms of **1** and the tridachione precursor **2**. **c** Mass spectrum of **1**. **d** Mass spectrum of tridachione precursor **2**.

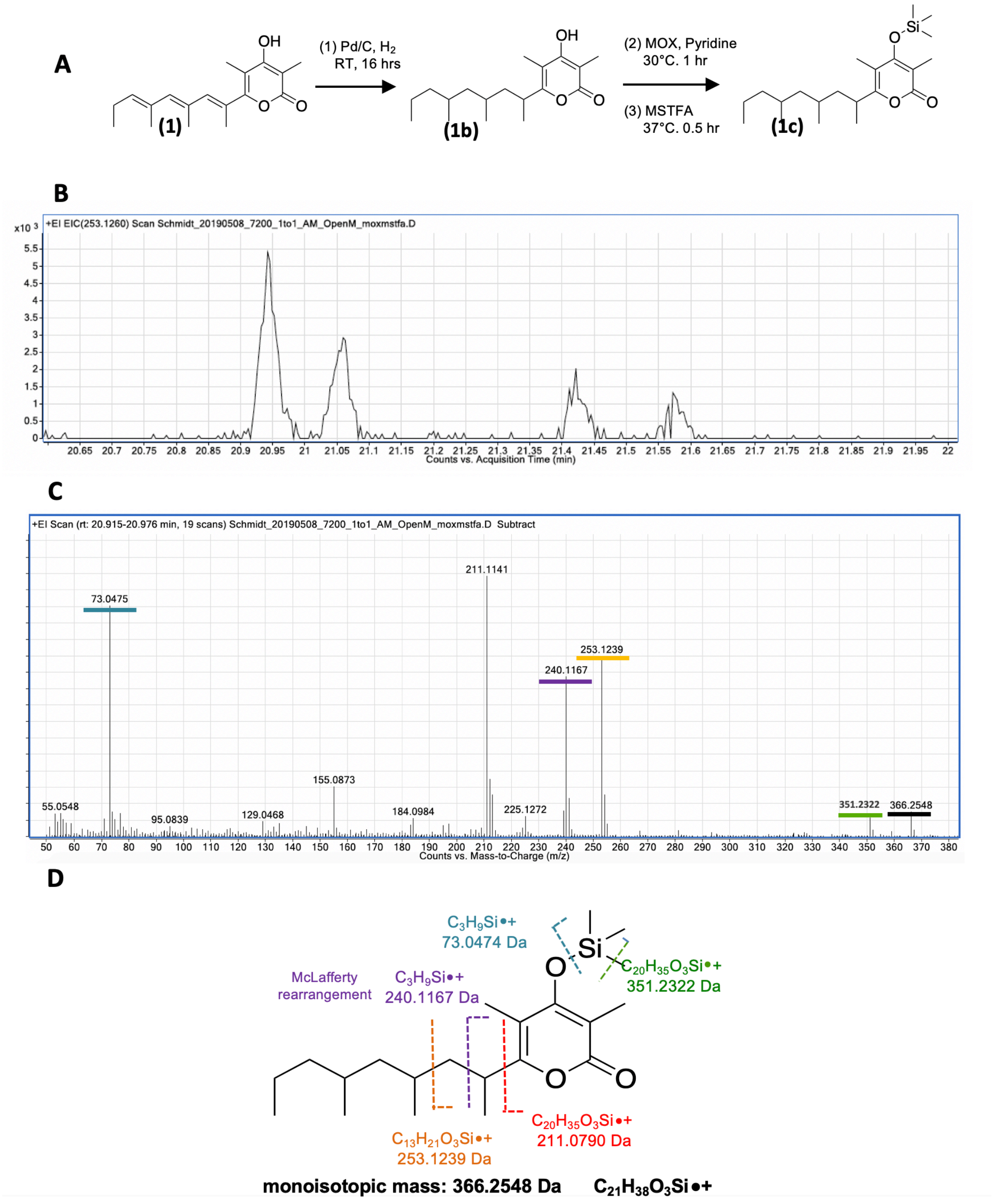

Supplementary Figure 5. Chemical characterization of compound 1 by GCMS. a Schematic of the hydrogenation and derivatization reactions of **1** prior to GCMS analysis. Pd/C: 10% palladium on carbon; MOX: *O*-methoxylamine hydrochloride; MSTFA: *N*-methyl-*N*-trimethylsilyltrifluoracetamide. **b** Extracted ion chromatogram of compound **1c**, showing all four

possible isomers eluting from the GC. **c** MS/MS spectrum from the first peak of **1c** observed at t = 20.95 min. All four isomers have nearly identical MS/MS spectra. **d** Schematic of the assigned fragmentation pattern of **1c**, showing the predicted *m/z* for each fragment. In addition to the assigned ions shown here, the major fragment at 240.1167 is readily explained by a McLafferty rearrangement.

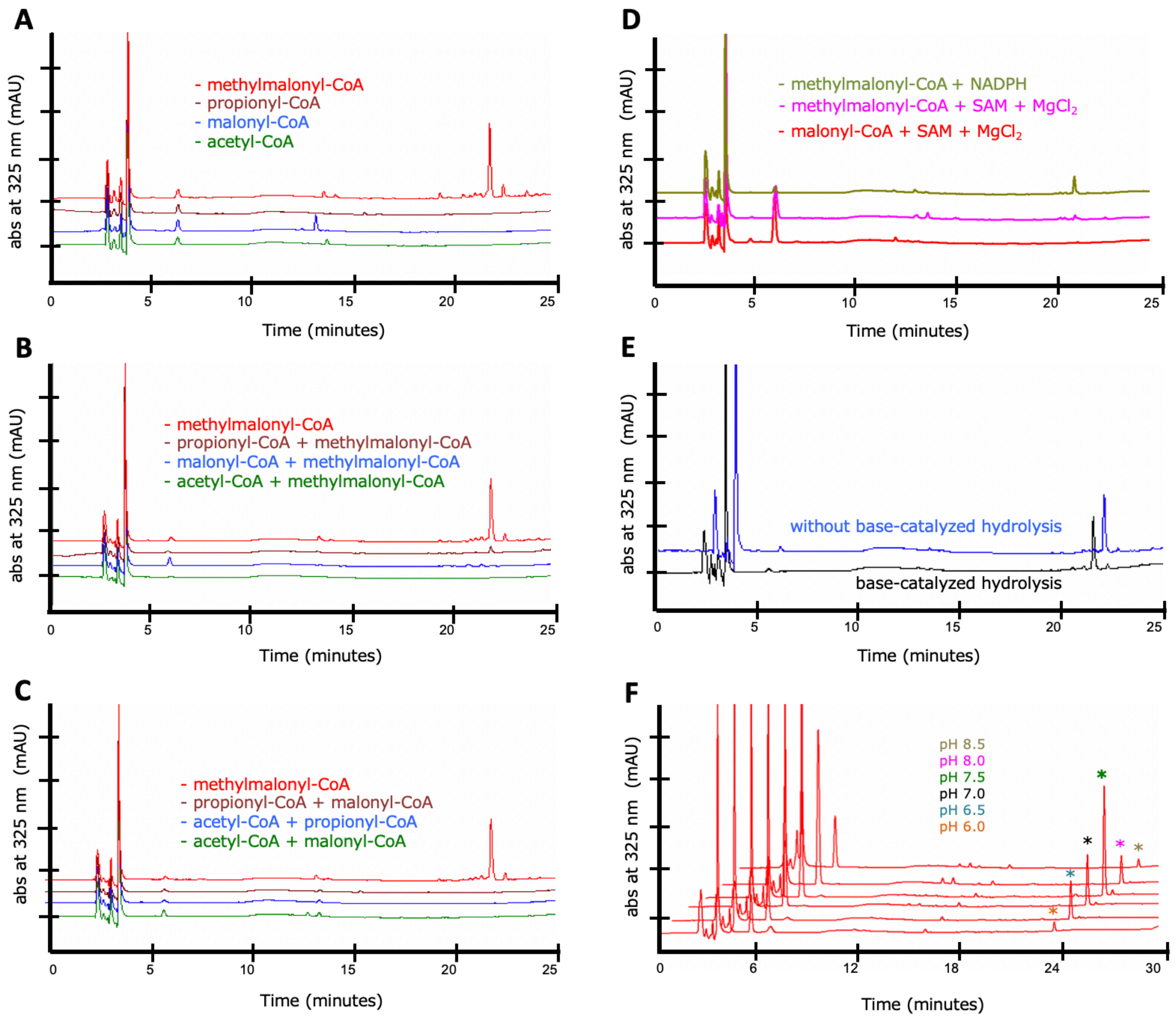

Supplementary Figure 6. **Biochemical characterization of EcPKS1.** Substrate specificity assay of EcPKS1 (A-C)**.** Each figure represents biochemical assays using EcPKS1. Following assay, an aliquot was injected and analyzed by DAD-HPLC. Each different colored trace indicates an assay run with different substrates as shown by color coding. In all cases, a product is only seen using methylmalonyl-CoA, at $t \sim 22$ min. Note that 2 mM NADPH is also present in all reactions analyzed in this figure. **a** EcPKS1 incubated with single acyl-CoA as starter and extender units. **b** EcPKS1 incubated with methylmalonyl-CoA and putative starter unit acyl-CoA substrates. **c** EcPKS1 incubated with mixed acyl-CoA substrates as starter and extender units. **d** Functional analysis of EcPKS1 methyltransferase domain**. e** EcPKS1 enzymatic reaction

containing SAM, $MgCl_2$, NADPH and malonly-CoA as substrate did not yield new products, suggesting that the MT domain of EcPKS1 is inactive. Base hydrolysis of EcPKS1 reaction did not increase product yield**.** An HPLC-DAD analysis of enzymatic reactions using methylmalonyl-CoA with and without a final addition of sodium hydroxide, showing that the yield is virtually identical in both conditions. **F** Optimization of enzyme assay**.** Using EcPKS1 with methylmalonyl-CoA, NADPH, and the buffer conditions described in Methods, the pH was varied, showing optimum pyrone formation at pH 7.0. All *y*-axes indicate monitoring at λ 325 nm, while all *x*-axes show the elution time in minutes.

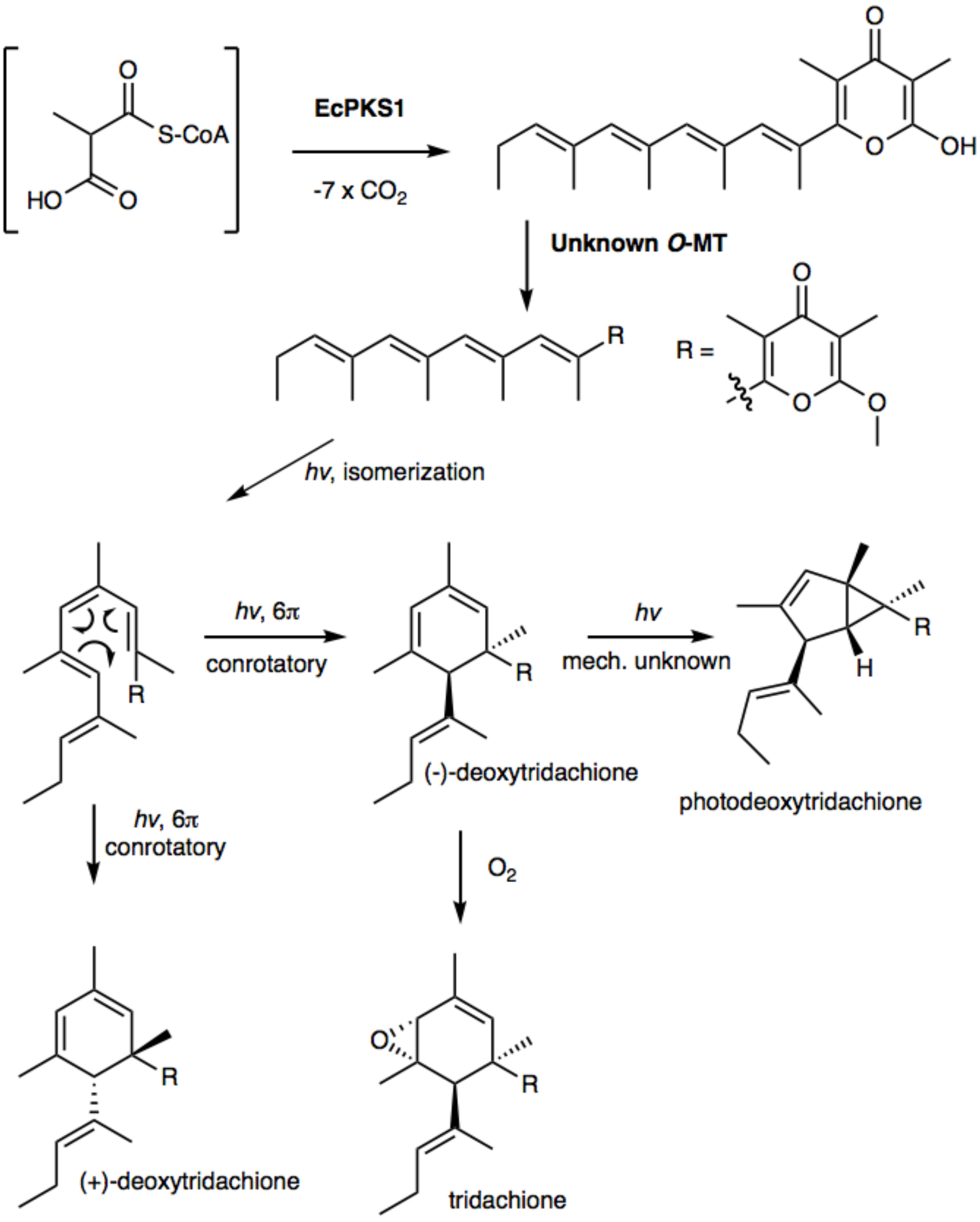

Supplementary Figure 7. Proposed biogenesis of sacoglossan polypropionates. Previously, feeding studies demonstrated that algal kleptoplasts convert CO_2 into fixed carbon, which is incorporated into tridachione and relatives. Here, we show that EcPKS1 synthesizes the tridachione precursor **2** using NADPH and methylmalonyl-CoA as substrates. The next biochemical step would involve methylation by an as-yet unidentified *O*-methyltransferase.

Previous chemical studies show that the linear, methylated compound **3** undergoes a series of photochemical cyclization and oxidation reactions to yield the structurally diverse sacoglossan polypropionates found in nature. Here, we show as an example the detailed mechanism leading to the known tridachione series. Note that the conversion of (-)-deoxytridachione into photodeoxytridachione has been performed synthetically under UV irradiation, with the proposal that the intermediate is a triplet diradical.

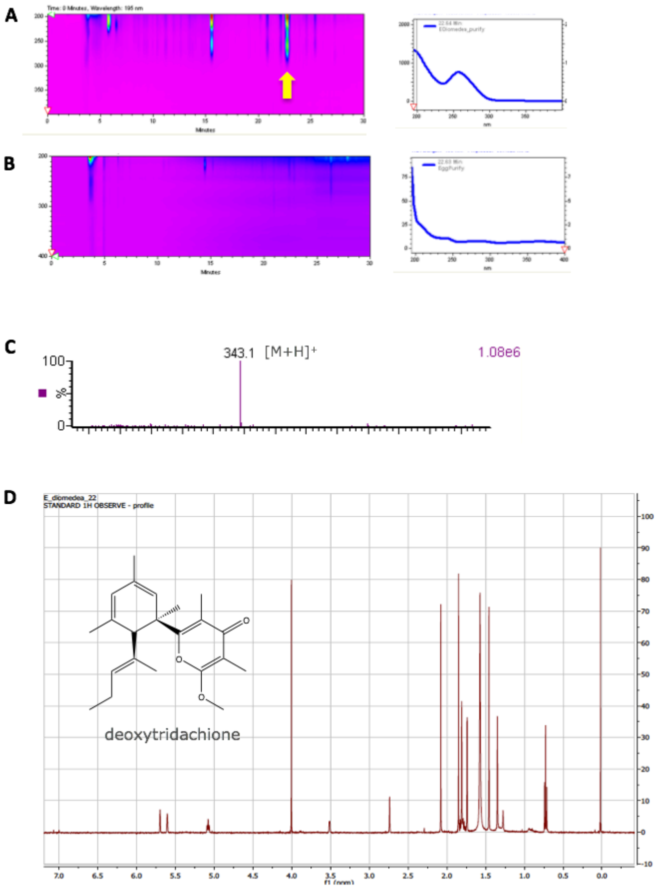

Supplementary Figure 8. Polypropionates in sacoglossans. Polypropionates are found in sacoglossans in the benthic slug stage. Specimens of adult *E. diomedea* were kept in an aquarium in Salt Lake City, in which they laid eggs. **a** HPLC profile of chemical extract from a tissue section of adult *E. diomedea* showed presence of many polypropionates. The major polypropionate (yellow arrow) is deoxytridachione**. b** Polypropionates are undetectable in extracts derived from egg ribbons of *E. diomedea*, indicating that polypropionates are specific to the adult. **c** Mass spectrum of purified deoxytridachione from *E. diomedea*. **d** ^{1}H NMR spectrum of deoxytridachione in $CDCl_3$.

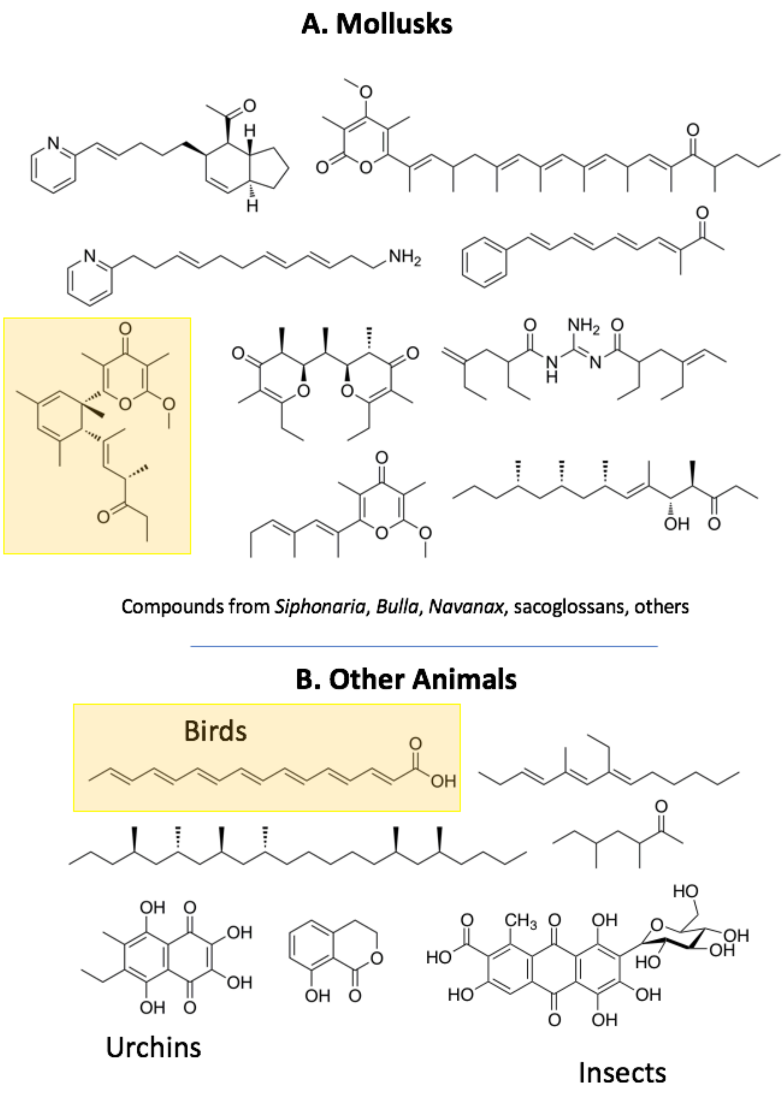

Supplementary Figure 9. Diverse PKS products from animals. These are representative of an enormous chemical diversity of polyketides from animals. Compounds shown are isolated from animals, but only those in yellow boxes indicate compounds that are firmly associated with animal genes or proteins. For other compounds, the genes and proteins involved are either unknown or they are not completely established via biochemical or complementation experiments. Nonetheless, based upon data in this paper and elsewhere, we propose that the compounds shown are likely compounds made by the animals. Further experimental work is required to firmly identify genes and proteins involved in the biosynthesis.

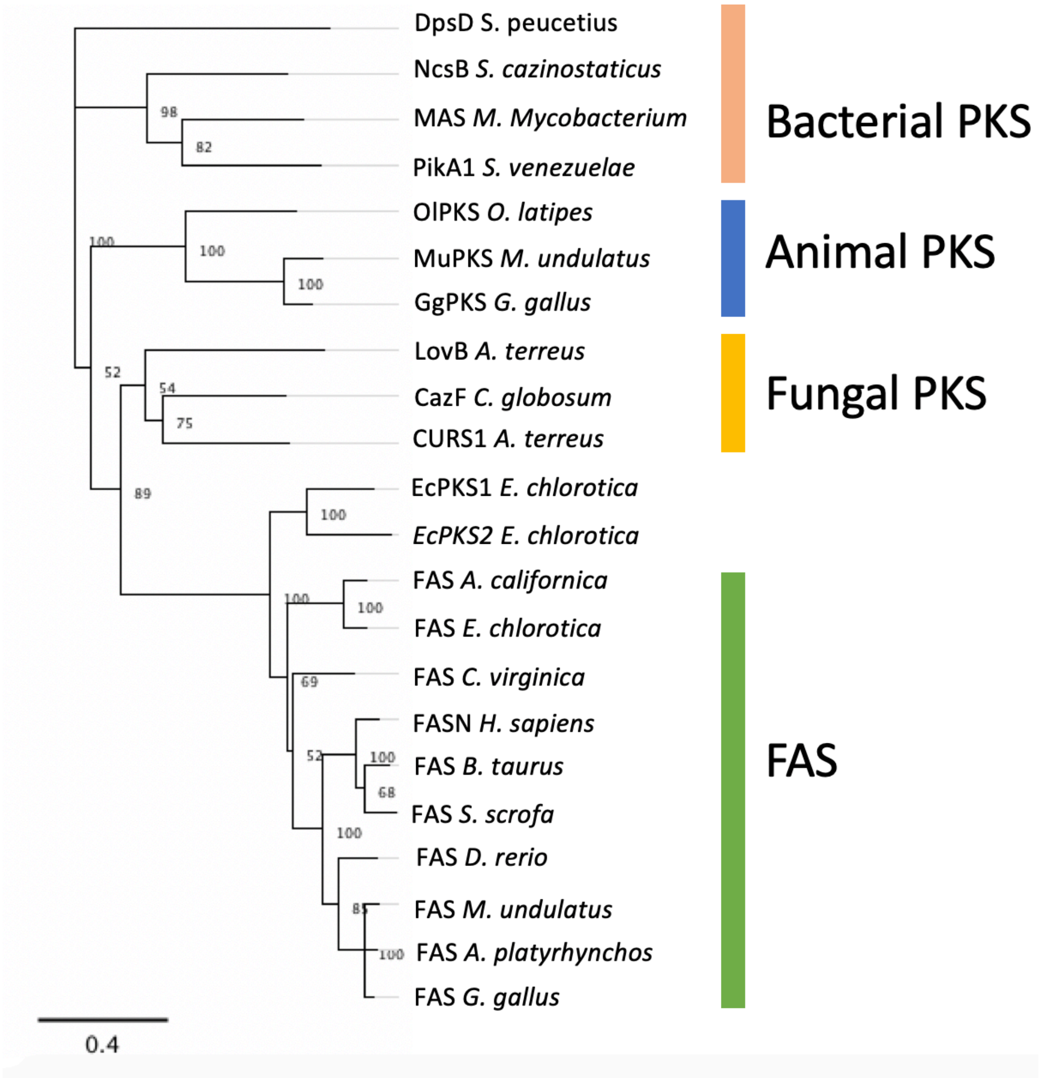

Supplementary Figure 10. Phlyogeny of acyltransferase domains in bacteria, fungi and animals. A phylogenetic tree based on alignment of acyltransferase domain (AT) of sacoglossan polyketide synthase with other known type 1 PKS and FAS from bacteria, fungi and higher metazoans. DpsA, a type II PKS from *Streptomyces peucetius* is used as an outgroup. This was created using the same method used for Fig. 6.

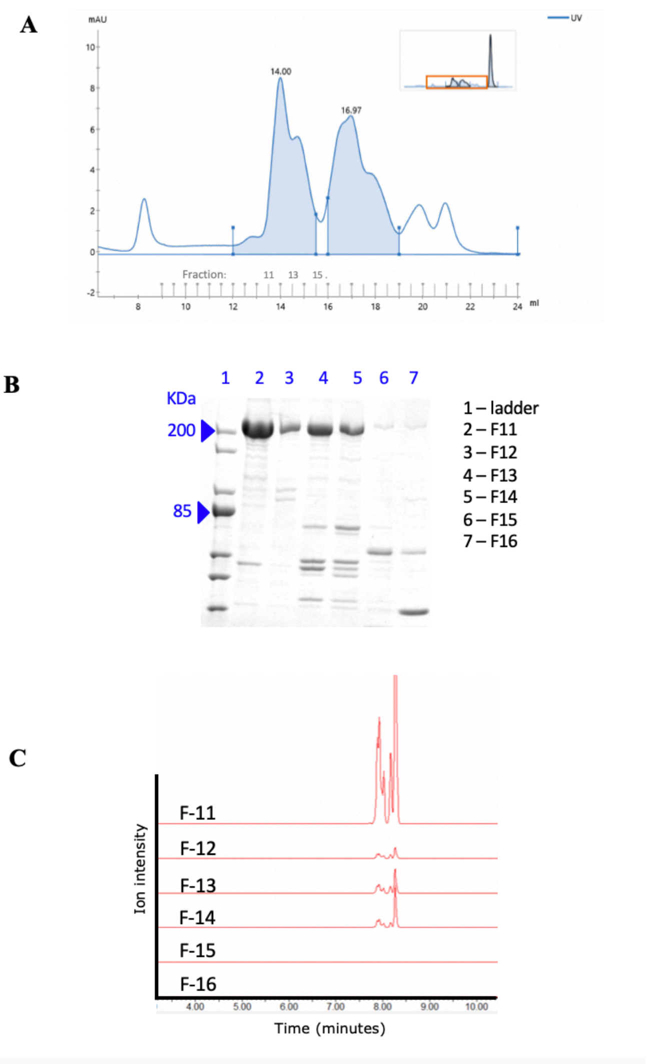

Supplementary Figure 11. Evaluation of EcPKS1 purity and activity by size exclusion chromatography. a SEC profile of EcPKS1 recovered from Ni-NTA purification. Collected fractions were concentrated and checked for purity by **b** SDS-PAGE and **c** enzyme activity through formation of compound (1) by LCMS. Comparing the activity of SEC collected fractions, we find that, formation of compound (1) is directly proportional to the concentration of EcPKS1. This shows that the enzymatic activity is from EcPKS1 and not from other minor contaminating proteins still present after purification. This experiment was performed twice to

further confirm results with Ni-NTA-purified proteins, which were performed in triplicate with three biological replicates.

MAPNNTQQEEASKGGNSSPEAYLPYSGDIAIAGISGRYPESDSVGEFRDNLFGKVNMLTCDDRRWKLGHLDLPDVLGKLRSVDRFDSEFFNLNSKQTEMLDPQTRLLLEVCYEAIVD
AGESLASVKGSRTGMYLAISSSEPEQAWICRQDPYIVMGCPHTMSPNRISFFFDLHGPSIAYDTACSSVLVALEAAFQHMRTGVIDSAIVAGVNTCFRALTSKVYQNMGMLGPEAC
KAFDSSGNGYARSEIVSALFLKKSSDSKRIYCSVVNVKTNNDGFTPQGLTFPSGEIQEQLMRNVYADCKLNPKEVSYFECHGTGTPAGDPQETNAIYRVMCTPDKREPLLIGSVKSNM
GHAETGSAMASITKVILAMHEGFIAPNLHFRSPNPKIEGLRDGKMAVVTEATPWSGGYMAINSFGMGGSNAHAVLRSYDVSSSKPHPSAHKPRLFTYSARTEHGLRAILREAQTHA
ASMEFHALCQASADAPLGSMPYRGATILNGQHDFEVVEKCKSKAREVWFIYAGMGSQWVGMARCLMQLDVFRHSLEKSAAVLKPHGVDLLNILSEGTEDTLRTILNPFVCITSIQV
ALTDLLWSMGIRPDGIVGHSMGEVGCAYCDGCLTAEEAVLTAYWRGKCVTDGKVPPGKMAAVGLTWEEAKSQCPAGVVPACHNAEDSVTISGAADVMLKFMEELKAKDVFVRE
VNSSNIAYHSYFMENIYASLKDSLSKVISPKPRTARWLPTSVPEELWDSAPAQSSSAEFHANNLVSPVLFHEALQKIPPTAIAIELAPHGLLQSVIKRTLGNESVCVGLQKRNYADNLEF
LFASLGKCFANGLSLNPLACYPPVEFPVPKGTPRLSDMVAGAWDHSAQWLVPKNEDFEGRVQASGSDSSYSIDVSADSPDRYLLDHQVDGRELFPACGCLVLAWKTLAALNGRDF
EQMPVRLSRVEIHQAMFLPKSGSATVTVSVMPRTGEFQVCENENLLASGFVTCPDKDVLETSTHAQTRSSLQDRPATEVLTRDEVYRELILRGYEYGPYFQGILRASVDGQESEITW
DGRWVSFMDSVLQMDILARPGDYQMLPIKFQSINIDPRVQPAAPAEDEDVVVLPGRFDPVLDIVSAGGVEIRGLETISASRRLTHAPEVVEEYRFVPHHVTGRDDPGAKRPGATVDI
REYADACLAFAVQGIKKWLSEDKDKVLPQKDLLQDALGLANQDLGSKSSSSDFISAKAALERILKQQNGHQQHGFGLFHTLNLAFSEPLEIGFRETLKNKIHHMRYDMWDDCLMSA
VECADSLKLCIDTVAENTTSHIVNVLEAGAAKGAFYRRAIPEALAKFSGKDYRYTVGDASPMDDAKEFSVKTLQFDAYDPANFPASQAHAHDLLVLKWVLHQQEDLDAAMAGFCG
FVRPGGFILVQEFVHRLPTLLAVEAVTDHPLPRKSGDRVLGRYYSAAQWRELFRRHGLVEVIHRSDGALADMFLLRSRVEVMTPPTVLHLDDLSCSWLEEVKAKYSDLEAMPQDAR
LWLVGKSDCNGMLGFFNCLRQEPGSERVRCVQVCGDSVPDLSPGSAEFKYLAEMDLAFNVHKDGKWGVYRHLAITDDQRRQQFPTEHAFVDTLTSGDLSTLTWVRSPLNLHASS
EKGQDCELCTVYMAGVVSRDLALACGKLRRDELPAGMFCKEGTLGIEFSGRDTKGKRVMGLCAPPALASSVLCLRSSLWSVPQHWSLEEAATVPVAYSTAYYALVIRGHVRPGDTV
LVHAGGSPVGQAAIAVAQSCGCEIFISTATDAETSSLKSMFPRLKDRNFCSCKDASFERHVKKETSGKGVDIILNCTTGELLGASIRLLASRGRFLNLASGRGSDAELVFSGSGRRDTSF
HDINLDTLIDAQGPEWTELTSLVQKGIQSGLVKPLARTVYAMDRLVDVFKLLEEGAQAGKLLVKIREEEAEKITLPAKKTFEAVPRTFFHPAKSYVIVGGLGGFGLELAHWMVLRGVR
KLVLTSRNGITTGYQTRKIAFLRSLGADIVVCAVNVTSQAAADRLVKTATDLGPLGGVFNLGLNLRDALLVEQTAENYKQTLEAKIQTTSLLDGISRSPKIQPTLDHFVMFSSLSAGHGI
PGQTNYGWGNSYMDRLCEKRRAQGLPGLSIQWASIADVGFVGTKGNNVVIEGKWPQRMYNCLQVCDYFLSQNRPVVACHVLAEKVKAAVEGEETVGQQVIKAVGNVLGLKSV
SGVDPDKVFLDLGLDsLMSVEIKQMLERDLDLALGTKDIQMLTFAQLQAMVHHHHHH

Supplementary Figure 12. Proteomic analysis of EcPKS1. A tryptic digest of EcPKS1 was applied to HPLC-MS, and the resulting ions were searched using Mascot. Shown in red are regions of the protein that were positively identified in this experiment.

Supplementary References

1 Dawe, R. D. & Wright, J. L. C. The major polypropionate metabolites from the sacoglossan mollusc, *Elysia chlorotica*. *Tetrahedron Lett.* **27**, 2559-2562 (1986).

2 Ireland, C. M. & Faulkner, D. J. The metabolites of the marine molluscs *Tridachiella diomedea* and *Tridachia crispata*. *Tetrahedron* **37**, 233-240 (1981).

3 Diaz-Marrero, A. R., Cueto, M., D'Croz, L. & Darias, J. Validating an endoperoxide as a key intermediate in the biosynthesis of elysiapyrones. *Org. Lett.* **10**, 3057-3060 (2008).

4 Ireland, C. & Scheuer, P. J. Photosynthetic marine mollusks*: in vivo* ^{14}C incorporation into metabolites of the Sacoglossan *Placobranchus ocellatus*. *Science* **205**, 922-923 (1979).

5 Fu, X., Hong, S. P. & Schmitz, F. J. New polypropionate pyrones from the Philippine sacoglossan mollusc *Placobranchus ocellatus*. *Tetrahedron* **56**, 8989-8993 (2000).

6 Gavagnin, M., Spinella, A., Castelluccio, F. & Cimino, G. Polypropionates from the Mediterranean mollusk *Elysia timida*. *J. Nat. Prod.* **57**, 298-304 (1994).

7 Ksebati, M. B. & Schmitz, F. J. Tridachiapyrones: propionate-derived metabolites from the sacoglossan mollusk *Tridachia crispata*. *J. Org. Chem.* **50**, 5637-5642 (1985).

8 Cutignano, A., Cimino, G., Villani, G. & Fontana, A. Shaping the polypropionate biosynthesis in the solar-powered mollusc *Elysia viridis*. *ChemBioChem* **10**, 315-322 (2009).

9 Carbone, M., Muniain, C., Castelluccio, F., Ianicelli, O. & Gavagnin, M. First chemical study of the sacoglossan *Elysia patagonica*: Isolation of a γ-pyrone propionate hydroperoxide. *Biochem. Systematics Ecol.* **49**, 172-175 (2013).

10 Pierce, S. K. & Curtis, N. E. in *International Review of Cell and Molecular Biology* Vol. 293 (ed K. W. Jeon) 123-148 (Academic Press, 2012).

11 Vitteri, C. *Estudios experimentales de comportamiento en la babosa marina fotosyntética Elysia diomedea (Opisthobranchia: Sacoglossa) ante distintas condiciones de luz y variedad de dieta* B.S. thesis, Pontificia Universidad Católica del Ecuador, (2012).

12 Evertsen, J., Burghardt, I., Johnsen, G. & Wägele, H. Retention of functional chloroplasts in some sacoglossans from the Indo-Pacific and Mediterranean. *Mar. Biol.* **151**, 2159-2166 (2007).

13 Maeda, T. *et al.* Algivore or phototroph? Plakobranchus ocellatus (Gastropoda) continuously acquires kleptoplasts and nutrition from multiple algal species in nature. *PLoS One* **7**, e42024 (2012).

14 Laetz, E. M., Rühr, P. T., Bartolomaeus, T., Preisfeld, A. & Wägele, H. Examining the retention of functional kleptoplasts and digestive activity in sacoglossan sea slugs. *Org. Divers. Evol.* **17**, 87-99 (2017).

15 Schmitt, V. *et al.* Chloroplast incorporation and long-term photosynthetic performance through the life cycle in laboratory cultures of *Elysia timida* (Sacoglossa, Heterobranchia). (Sacoglossa, Heterobranchia). *Front. Zool.* **11**, 5 (2014).

16 Christa, G. *et al.* Phylogenetic evidence for multiple independent origins of functional kleptoplasty in Sacoglossa (Heterobranchia, Gastropoda). *Org. Divers. Evol.* **15**, 23-36 (2015).

17 Pierce, S. K., Curtis, N. E. & Middlebrooks, M. L. Sacoglossan sea slugs make routine use of photosynthesis by a variety of species-specific adaptations. *Invert. Biol.* **134**, 103-115 (2015).

18 Hinde, R. & Smith, D. C. The role of photosynthesis in the nutrition of the mollusc *Elysia viridis*. *Biol. J. Linnean Soc.* **7**, 161-171 (1975).

19 Cartaxana, P., Trampe, E., Kühl, M. & Cruz, S. Kleptoplast photosynthesis is nutritionally relevant in the sea slug *Elysia viridis*. *Sci. Rep.* **7**, 7714 (2017).

20 Evertsen, J. & Johnsen, G. In vivo and in vitro differences in chloroplast functionality in the two north Atlantic sacoglossans (Gastropoda, Opisthobranchia) *Placida dendritica* and *Elysia viridis*. *Mar. Biol.* **156**, 847-859 (2009).
